# Optical coherence tomography angiography findings in patients undergoing transcorneal electrical stimulation for treating retinitis pigmentosa

**DOI:** 10.1007/s00417-020-04963-7

**Published:** 2020-10-10

**Authors:** Olga Zabek, Hanna Camenzind Zuche, Ursula Müller, Hendrik P. N. Scholl, Annekatrin Rickmann, Maria della Volpe Waizel

**Affiliations:** 1 Department of Ophthalmology, University Hospital Basel, University of Basel, Basel, Switzerland; 2 Institute of Molecular and Clinical Ophthalmology Basel (IOB), Basel, Switzerland; 3 Eye Clinic, Knappschaft Hospital Sulzbach, Sulzbach, Saar Germany

**Keywords:** Optical coherence tomography angiography, Macular vessel density, Peripapillary microvasculature, Inherited retinal diseases, OkuStim, TES, Transcorneal electrical stimulation, Retinitis pigmentosa

## Abstract

**Purpose:**

Transcorneal electrical stimulation (TES) is a novel treatment approach for patients with retinitis pigmentosa (RP). The aim of our study was to observe changes in optical coherence tomography angiography (OCTA) that would be attributed to TES treatment.

**Methods:**

A total of 73 eyes were included: 43 eyes of 22 subjects (11 ♀, 11 ♂) suffering from RP were examined at baseline (BL), after first stimulation (TS), 1 week (1W), and 6 months (6M) after treatment initiation and were compared with 30 control eyes of 15 subjects (8 ♀, 7 ♂). TES was performed simultaneously on both eyes for 30 min weekly. OCTA scans of 9 × 15 mm were recorded with a PLEX Elite 9000 swept-source OCTA device (Carl Zeiss Meditec AG, Jena). Vascular density metrics such as perfusion density (PD) and vessel density (VD) were calculated automatically for the macular area by using standardised extended early treatment diabetic retinopathy study (ETDRS) grids centred around the fovea. In addition, the capillary perfusion density (CPD) and the capillary flux index (CFI) of the peripapillary nerve fibre layer microvasculature in all four quadrants of an annulus centred at the optic disc were measured. All parameters were determined over all retinal layers and separately for the superficial (SCP) and deep capillary plexus (DCP). ANOVA-based linear mixed-effects models were calculated with SPSS®.

**Results:**

Throughout the course of TES treatment, the macular VD and PD of all retinal layers in all subsections showed a slight decrement without reaching statistical significance, also when analysed separately in the SCP and DCP (*p* > 0.08). In analogy, the average CPD and CFI also presented with a slight decrement (*p* > 0.20). However, when compared with controls, most OCTA parameters showed a significant decrement (*p* < 0.05). When analysed systematically in all subsections of the extended ETDRS grid, the temporal macular subsections within the outer ring (radius 1.5–3 mm) and also of the peripheral C1, C2, and C3 rings (radius 3–7.5 mm) showed lower VD and PD values when compared with the other subsections (*p* < 0.05).

**Conclusion:**

Vascular density metrics in the macular region and the peripapillary microvasculature appear to remain unaffected by continuous TES treatment within a period of 6 months.

**Electronic supplementary material:**

The online version of this article (10.1007/s00417-020-04963-7) contains supplementary material, which is available to authorized users.

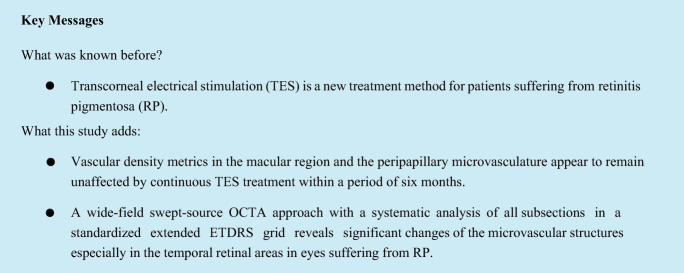



## Introduction

Transcorneal electrical stimulation (TES) is a new treatment method for patients suffering from retinitis pigmentosa (RP) [1, 2]. The pathophysiological mechanisms are not fully understood. However, it is likely that the stimulation induces the release of neurotrophic factors that improve the remaining retinal cells’ survival [2–9]. A more detailed explanation is given in the TES pivotal trial and in the TES study presenting 1-year results [1, 2].

In order to evaluate the therapeutic TES response in RP patients in a short-lasting, non-invasive, contact-free, and patient-friendly way, a recent study has shown that retinal vessel oximetry (RO) might be superior over full-field electroretinography (ffERG) or visual field (VF) as outcome measurement [1–3]. However, since RO devices so far are mainly used in a scientific context and thus are mostly preserved to specialised ophthalmology centres, further monitoring methods that are short-lasting, non-invasive, contact-free, and patient-friendly and have a better availability in daily clinical practice are needed.

It is known that changes of the retinal vasculature with vessel attenuation are a hallmark of RP [4–11]. In this context, swept-source coherence tomography angiography (OCTA) that allows for a non-invasive depth-resolved visualisation of the retinal and choroidal microvasculature in vivo might be useful as a monitoring method for TES treatment.

Several recent studies conducted with OCTA have reported a reduction of retinal vessel density and retinal perfusion density, impaired choroidal blood flow, or significantly altered sizes of the foveal avascular zones in eyes of patients suffering from RP [12–27]. Given the known microvascular alterations in RP, it might be beneficial to use OCTA in the context of TES treatment.

Furthermore, so far, many studies either used small areas for OCTA analysis (3 × 3 mm or 6 × 6 mm) that mainly focused only on parafoveal macular density metrics and/or presented these results as averaged values and not systematically in standardised segmentation grids over a wide-field area that covers the whole macula and also the peripapillary regions.

Therefore, the aim of our study was to compare OCTA parameters in RP patients undergoing TES therapy in order to find changes that might be attributed to TES treatment. Furthermore, we aimed to use wide-field swept-source OCTA in order to perform the analysis of OCTA parameters systematically within all subsections of a standardised extended ETDRS grid.

## Methods

This prospective observational study was conducted from January 2018 until December 2018 in a single ophthalmology centre (University of Basel, Department of Ophthalmology, Switzerland) on a total of 73 eyes: 43 eyes from 22 subjects suffering from RP were compared with 30 eyes of 15 healthy controls. Approval of the local authorities (Ethics Commission of Central and Northern Switzerland, EKNZ, Basel, Switzerland) was obtained with a positive vote for prospective observational investigation (trial number EKNZ BASEC 2017-00937).

The inclusion criteria for all study participants were Caucasian origin, refractive spherical equivalent error of < 6 dioptres for either myopia or hyperopia, no previous ocular surgery, implants as pacemakers, or other ocular or systemic pathology (intraocular surgeries such as vitreoretinal surgery, diabetic retinopathy, retinal or choroidal neovascularisation, exudative age-related macular degeneration, glaucoma, history of retinal detachment, diseases of the optic nerve, systemic diseases such as diabetes mellitus, systemic hypertension, or neurological diseases) that may influence the OCTA data. In addition, the inclusion criteria for all patients were clinical and electrophysiological diagnosis of RP, absence of macular oedema, a residual central VF of > 10 degree visual angle, and no contraindications for TES therapy. Further exclusion criteria were OCTA scans with inadequate quality (signal strength index (SSI) < 8) due to significant motion artefact or incorrect automatic segmentation, unreliable performance of TES therapy, absence of an electrical stimulation threshold (expected in advanced or severe RP), and expressed unwillingness to participate in the study. The research procedures were performed in accordance with institutional guidelines and the Declaration of Helsinki. Written informed consent was obtained before examination. All healthy participants received an ophthalmic examination including refraction, best-corrected visual acuity (BCVA with standardised ETDRS charts), slit lamp examination with biomicroscopy, and funduscopy. All patients underwent a detailed ophthalmic examination at baseline (BL) and after 6 months (6M) of TES treatment that included refraction, best-corrected visual acuity, slit lamp examination, biomicroscopy, funduscopy, fundus autofluorescence, and VF with semi-automated kinetic perimetry (V4e, III4e, I4e, III3e isopters tested with Octopus 900®, Haag-Streit AG Bern, Switzerland), if supported from the patient’s health insurance including molecular genetic assessment and full-field electroretinography (ffERG, Diagnosys LLC Espion system; ISCEV standard [28]). In cases where the ffERG was extinguished at BL, it was not repeated at the 6M follow-up. The follow-up interval was chosen because of the leasing policy of the Swiss TES dealer that provided a trial period with reduced fees for 6 months. All patients were recruited from the hospital’s hereditary retinal degeneration consultation hour and were diagnosed by one experienced fellowship-trained retina specialist (HPNS).

Prior to OCTA measurements, both pupils were dilated with Tropiphen eye drops. This medication is prepared in our institutional pharmacy as a combination of tropicamide 0.5% and phenylephrine 1%. Three drops at 10-min intervals were applied per eye.

### Transcorneal electrical stimulation

TES (OkuStim®, Retina Implant, Reutlingen, Germany) was performed according to the recommendations of the pivotal trial [1] and the study presenting 1-year results measured with VF and electroretinography [2]. The OkuStim® system has three parts: the stimulation box, a special frame that is adjusted to the patient’s face, and electrodes that have to be placed into the frame to ensure good contact with the conjunctival tissue of the lower eyelid and the inferior bulbar conjunctiva for a low impedance during TES threshold measurement and stimulation. Standard DTL-based electrodes were used with an additional stirrup in order to simplify a stable position in the frame. In addition, a ground red-dot electrode (3M Europe, Diegem, Belgium) is attached to the ipsilateral side of the forehead. The OkuStim TES system is able to deliver different stimulus intensities on both eyes simultaneously. All patients received a determination of their individual electrical phosphene thresholds (EPTs) in a darkened room on each eye separately in three independent measurements per eye by one single experienced operator (UM). The individual stimulation parameters were programmed onto a patient’s individual USB stick required to start stimulation by plugging it into the OkuStim TES system. Stimulation was performed once per week for 30 min at 200% of the EPT simultaneously on both eyes at 20 Hz with current-balanced 5-ms positive deflections followed by 5-ms negative deflections. Every patient could choose between a supervised TES stimulation in the hospital or a non-supervised TES stimulation at home. However, the latter required a thorough instruction of the patient or a relative of her/him in order to perform a reliable and safe TES stimulation. For security reasons, the OkuStim box is programmed to stop stimulation automatically in case the impedance increases. In these cases, or if the handling is not reliable enough to ensure good tissue contact, patients were scheduled for additional instruction appointments. For all patients, TES stimulation parameters (duration, timing, impedance, and frequency of all stimulation sessions) were recorded and reviewed for consistency before inclusion in the study.

### Optical coherence tomography angiography acquisition

OCTA scans of 9 × 15 mm centred around the fovea were performed at the baseline visit before TES initiation (BL), shortly after EPT determination including the first TES stimulation (ST), as well as 1 week after the first TES stimulation (1W) and at the 6-month follow-up (6M). We used a PLEX Elite 9000 swept-source OCTA device (Carl Zeiss Meditec AG, Jena) with an active eye-tracking system that assesses simultaneously the fundus and the OCTA image acquisition in order to achieve a better signal-to-noise ratio. All scans used for the analysis were anonymised and calculated automatically in the Zeiss© ARI network environment, using the newest versions of the macular density algorithm (v0.7.2) and the peripapillary nerve fibre layer microvasculature density algorithm (v0.9). All parameters were determined over all retinal layers (retina slab) and separately for the superficial (SCP) and deep capillary plexus (DCP). All OCTA scans were checked independently by two retina specialist (MdVW and AR) to ascertain a correct position of the subsection grids for all macular and peripapillary analyses. Furthermore, the automated segmentation of the retinal layers used for the analysis of the SCP and DCP was reviewed for correctness as well. The OCTA device provides an automatic segmentation of three depth-resolved slabs: the retinal slab measured over all retinal layers, the SCP between the internal limiting membrane (ILM) and the inner plexiform layer (IPL), and the DCP between IPL and the outer plexiform layer (OPL).

#### OCTA analysis of the macular region

**Fig. 1:**
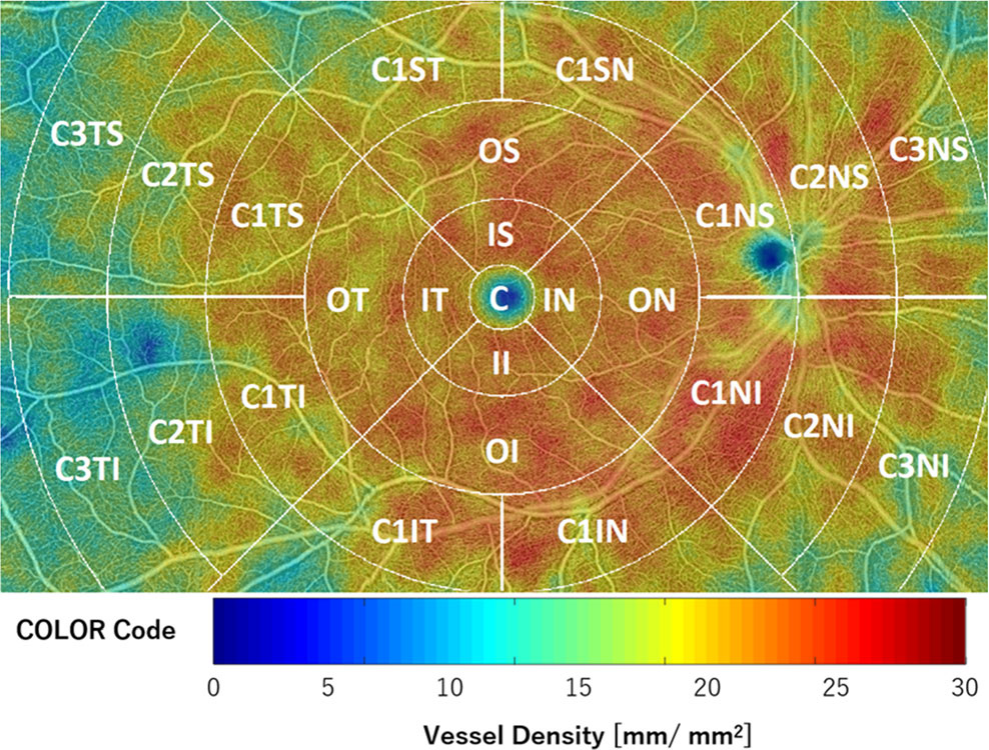
Example for a vessel density (VD) calculation within different sectors of a standardised extended ETDRS grid on a retinal OCTA slab in the right eye of a healthy subject. The colour bar represents the VD measurements as a colour code in mm/mm^2^. Results of the VD analysis are averaged values for both the entire scan area, but also for subsections of the ETDRS grid in different regions of measurement: (C) centrally in the foveal area within 1-mm diameter, (I) within an inner ring with a radius of 0.5–1.5 mm divided in four quadrants ((IS) superior, (II) inferior, (IT) temporal, and (IN) nasal quadrant), (O) within an outer ring of 1.5–3-mm radius divided in four quadrants ((OS) superior, (OI) inferior, (OT) temporal, and (ON) nasal quadrant). In addition, further subsections in extended rings C1 (radius 3–4.5 mm), C2 (radius 4.5–6 mm), and C3 (radius 6–7.5 mm) are calculated as well, using the labels (S) superior, (I) inferior, (T) temporal, and (N) nasal

Macular density metrics such as vessel density (VD) and perfusion density (PD) were calculated using standardised extended ETDRS grids in the macular area (Fig. 1). VD was defined as the total length of perfused microvasculature per unit area in the measurement region and is measured in mm/mm^2^. The result is a number with a minimum of 0 (no vessels) and an unbounded maximum. PD was defined as the total area of perfused microvasculature per unit area in the region of measurement. The result is a number that ranges from 0 (no perfusion) to 1 (full perfusion). The macular density algorithm uses binarised slab images of the whole retina, and separately the SCP and the DCP, the latter being cleared from projection artefacts. Large vessels are removed when quantifying all macular density metrics. The presented results are averaged density values for both the entire scan area and for subsections of the extended ETDRS grid in different regions of measurement: (C) centrally in the foveal area within 1-mm diameter, (I) within an inner ring with a radius of 0.5–1.5 mm divided in four quadrants ((IS) superior, (II) inferior, (IT) temporal, and (IN) nasal quadrant), (O) within an outer ring of 1.5–3-mm radius divided in four quadrants ((OS) superior, (OI) inferior, (OT) temporal, and (ON) nasal quadrant). In addition, since the 9 × 15-mm wide-field OCTA scans provide a relatively big area of measurement, further subsections in extended rings C1 (radius 3–4.5 mm), C2 (radius 4.5–6 mm), and C3 (radius 6–7.5 mm) were calculated as well, using the labels (S) superior, (I) inferior, (T) temporal, and (N) nasal as indicated in Fig. 1.

#### OCTA analysis of the peripapillary region

**Fig. 2:**
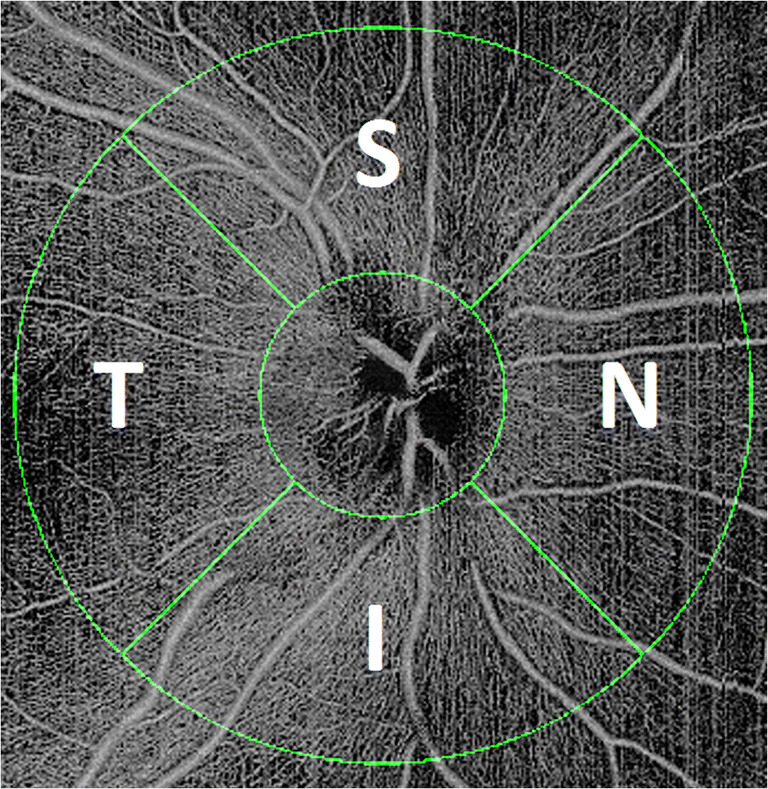
Example for the calculation of peripapillary microvasculature parameters in the right eye of a healthy subject. An optic disc–centred image protocol is applied, where two concentric rings are created in the peripapillary area: one with a diameter of 2 mm and second with a diameter of 6 mm. The region between these two circles defined the area of interest, in which all calculations were automatically performed. The results are presented as averaged values within four different sectors: (S) superior, (N) nasal, (I) inferior, and (T) temporal

Peripapillary microvasculature metrics such as the capillary perfusion density (CPD) and the capillary flux index (CFI) of the peripapillary nerve fibre layer microvasculature were measured. CPD was defined as the total area of perfused microvasculature per unit area in a region of measurement. The result is a number ranging from 0 (no perfusion) to 1 (fully perfused). CFI was defined as the total weighted area of perfused microvasculature per unit area in a region of measurement. The peripapillary microvasculature algorithm performs a segmentation of the ILM and retina nerve fibre layer (RNFL), creates radial peripapillary capillary (RPC) vasculature enface, and calculates capillary density metrics over an annulus centred at the optic disc. Large vessels are removed when quantifying the microcirculation. An optic disc–centred image protocol was applied, where two concentric rings were created in the peripapillary area: one with a diameter of 2 mm and second with a diameter of 6 mm. The region between these two circles defined the area of interest, in which all calculations were automatically performed. The results are presented as averaged values within four different sectors: (S) superior, (N) nasal, (I) inferior, and (T) temporal (as labelled in Fig. 2).

### Statistical analysis

Study endpoints were the VD (mm/mm^2^) and the PD (no unit) in the macular region, as well as the CPD (no unit) and the CFI (no unit) in the peripapillary region at BL, after the ST, 1W, and 6M after TES.

For statistical evaluation, normal distribution for all parameters was ensured with histograms and Shapiro–Wilk tests. Since RP may be presented asymmetrically or even unilaterally [29, 30], we calculated ANOVA-based linear mixed-effects models with SPSS® (IBM SPSS Statistics®, version 22.0.0.0) which allows taking the dependency of the left and right eyes in the same subject into account and its suitability for repeated measurements. All pairwise comparisons with ANOVA were adjusted with Bonferroni corrections. The results are presented as arithmetic mean and standard deviation (± SD) for all examined groups, with their corresponding *p* values. To calculate the effect of TES on OCTA measurements at the different follow-up visits (BL, ST, 1W, 6M), the eye, the refractive spherical equivalent, and the follow-up effect were taken into account, where the eye, refraction, and the follow-up were treated as fixed factors and the subject as a random factor. All results are presented as mean and standard deviation (± SD) for all examined time points and subsections with their corresponding *p* values. A *p* value of < 0.05 was defined as statistically significant.

## Results

Altogether, 73 eyes were enrolled in the study: 43 eyes of 22 patients diagnosed with RP (11 ♀, 11 ♂; 22 OS, 21 OD, 47.5 ± 15.2 years, range 20–81 years) and 30 age-matched control eyes from 15 healthy participants (8 ♀, 7 ♂; 15 OS, 15 OD, 43.8 ± 16.6 years, range 21–71 years). Twenty patients decided to perform a non-supervised TES stimulation at home after having received a thorough instruction; 2 patients received supervised TES stimulations in the hospital. A subset of patients was screened for mutations in retinal disease genes, and mutations in the following genes were found: three cases with USH2A; three cases with EYS; one case each with USH3A (CLRN1), DHX38, RHO, RPGR, and RP9. In controls, the mean logMAR BCVA was 0.01 ± 0.07. In all patients, the mean logMAR BCVA at BL was 0.23 ± 0.25 and improved slightly after 6 months of TES treatment (6M) to 0.22 ± 0.22, however without reaching statistical significance (*p* = 0.30). The mean VF, measured with V4e isopter area, was 4470.2 ± 4318.2 deg^2^ at BL and worsened to 4351.5 ± 4437.8 deg^2^ at the 6M follow-up (*p* = 0.41). No OCTA parameter showed a statistically significant influence by gender, spherical equivalent, or the side of the eye (all *p* > 0.83).

### Macular microvasculature metrics

#### Comparison of macular VD and PD at baseline, after first stimulation, 1 week, and 6 months after TES and versus control

**Fig. 3:**
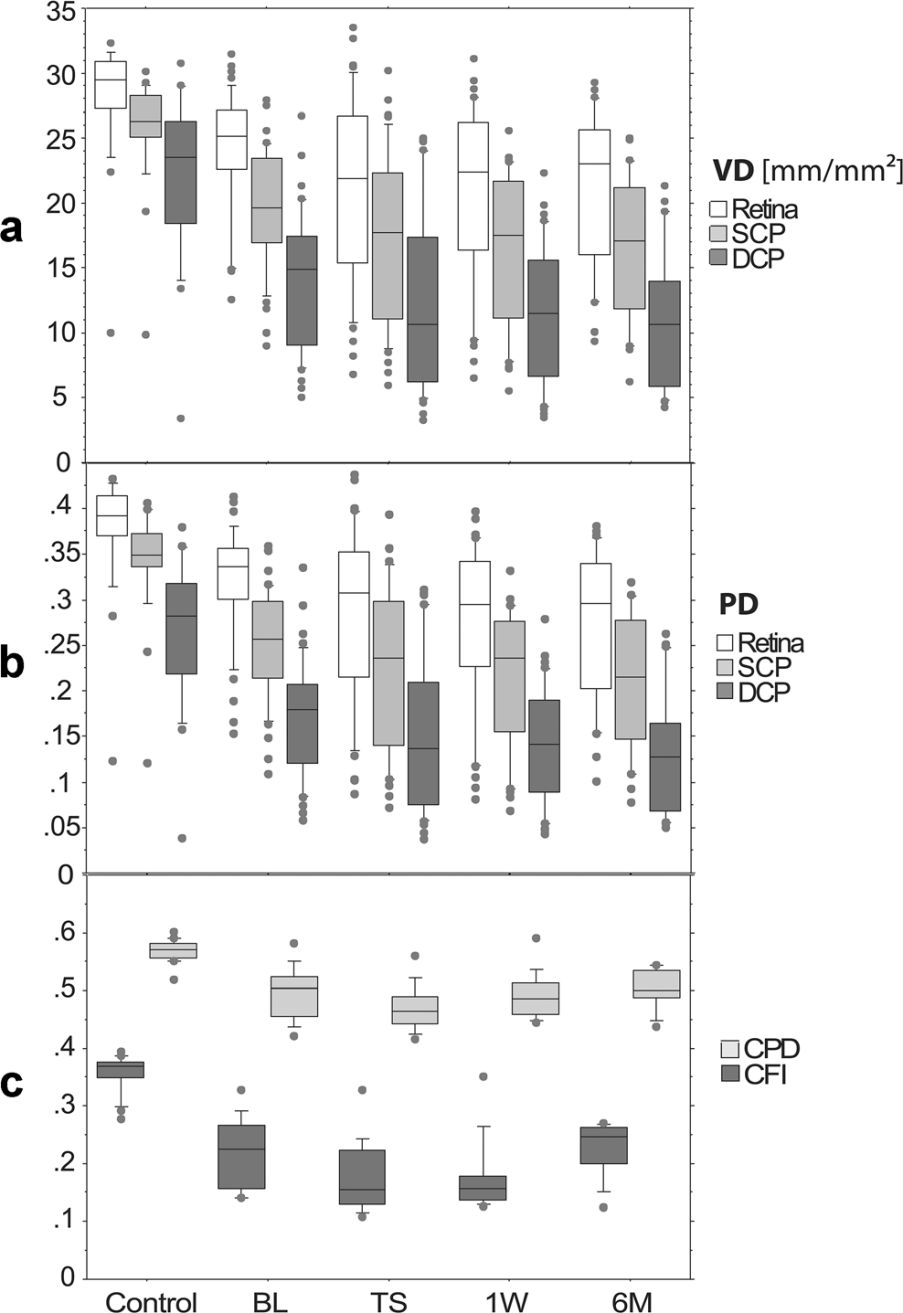
A, B Box plot analysis illustrates the average vessel density (VD, A) and average perfusion density (PD, B) of all retinal layers (retina slab, white), SCP (light grey), and DCP (dark grey). The *y*-axis shows the VD in mm/mm^2^, and the PD without unit for all follow-up visits (BL, TS, 1W, 6M) and control eyes are shown on the *x*-axis. C Box plot analysis illustrates the average capillary perfusion density (CPD, light grey) and the average capillary flux index (CFI, dark grey). The *y*-axis shows the CPD and CFI without units for all follow-up visits (BL, TS, 1W, 6M), and control eyes are shown on the *x*-axis. The upper and lower whiskers of the box plots present the minimum and maximum value, the upper and lower boarders of the box itself represent the 25th and 75th percentile, and the black horizontal bar within the box represents the median. The grey points indicate outliers

In general, the macular VD and PD parameters in all retinal layers, also when analysed separately in the SCP and DCP, were significantly lower in almost all subsections when compared with controls (*p* < 0.05, Tables 1, 2, and 3). However, in RP patients, these values presented only with a slight decrement without reaching statistical significance throughout the course of TES treatment: the average VD was 24.13 ± 4.71 mm/mm^2^ at BL, 21.15 ± 7.09 mm/mm^2^ at TS (*p* = 0.13), 20.71 ± 6.96 mm/mm^2^ at 1W after stimulation (*p* = 0.08), and 20.90 ± 5.88 mm/mm^2^ at 6M of TES stimulation (*p* = 0.17, Table 1, Table 2, Fig. 3). Similar observations were made when analysed separately in the SCP and DCP.

**Table 1:** Average vessel density (VD) and average perfusion density (PD) results for all retinal OCTA slabs (retina, SCP, and DCP), as well as the average peripapillary capillary flux index (CFI) and average capillary perfusion density (CPD) results for control eyes as well as for RP eyes with all follow-up visits (BL, TS, 1W, 6M) and including the corresponding *p* value in brackets. All presented *p* values correspond to the comparison versus baseline (upper value) or versus controls (lower value, italic notation); significant *p* values are bold and marked with an asterisk

Follow-up visit	VD retina (mm/mm^2^; mean ± SD)	VD SCP (mm/mm^2^; mean ± SD)	VD DCP (mm/mm^2^; mean ± SD)	PD retina (mean ± SD)	PD SCP (mean ± SD)	PD DCP (mean ± SD)	CFI (mean ± SD)	CPD (mean ± SD)
Control eyes	28.24 ± 4.63	25.63 ± 4.12	21.96 ± 6.32	0.38 ± 0.06	0.34 ± 0.06	0.27 ± 0.08	0.36 ± 0.03	0.57 ± 0.02
BL	24.13 ± 4.71 (p = 0.0160*)	19.60 ± 4.55 (p = 0.0001*)	13.94 ± 5.11 (p = 0.0002*)	0.32 ± 0.06 (p = 0.0029*)	0.26 ± 0.06 (p < 0.0001*)	0.17 ± 0.06 (p < 0.0001*)	0.22 ± 0.06 (p < 0.0001*)	0.50 ± 0.05 (p < 0.0001*)
TS	21.15 ± 7.09	17.07 ± 6.67	12.29 ± 6.87	0.28 ± 0.10	0.22 ± 0.09	0.15 ± 0.08	0.18 ± 0.06	0.47 ± 0.04
	(*p* = 0.1344) (p = 0.0008*)	(*p* = 0.1828) (p < 0.0001*)	(*p* = 0.5619) (p < 0.0001*)	(*p* = 0.1355) (p = 0.0010*)	(p = 0.1355) (p < 0.0001*)	(*p* = 0.5879) (p < 0.0001*)	(*p* = 0.2937) (p < 0.0001*)	(*p* = 0.4493) (p < 0.0001*)
1W	20.71 ± 6.96	16.53 ± 5.93	11.67 ± 5.28	0.28 ± 0.09	0.21 ± 0.08	0.14 ± 0.06	0.17 ± 0.06	0.49 ± 0.04
	(*p* = 0.0780) (p = 0.0002*)	(*p* = 0.0849) (p < 0.0001*)	(*p* = 0.3045) (p < 0.0001*)	(*p* = 0.0687) (p = 0.0010*)	(*p* = 0.0897) (p < 0.0001*)	(*p* = 0.3010) (p < 0.0001*)	(*p* = 0.1945) (p < 0.0001*)	(*p* = 0.9692) (p < 0.0001*)
6M	20.90 ± 5.88	16.57 ± 5.36	10.93 ± 5.28	0.27 ± 0.08	0.21 ± 0.07	0.13 ± 0.07	0.23 ± 0.05	0.50 ± 0.03
	(*p* = 0.1657) (p < 0.0001*)	(*p* = 0.1473) (p < 0.0001*)	(*p* = 0.1554) (p < 0.0001*)	(*p* = 0.0544) (p < 0.0001*)	(*p* = 0.0639) (p < 0.0001*)	(*p* = 0.1087) (p < 0.0001*)	(*p* = 0.9899) (p < 0.0001*)	(*p* = 0.9816) (p = 0.0001*)

**Table 2:** Results of the vessel density (VD) analysis for all retinal OCTA slabs (retina, SCP, and DCP) are presented as *p* values (ANOVA) between all four follow-up visits (BL, TS, 1W, 6M) and all subsections as indicated with the extended ETDRS grid in Fig. 1. Significant *p* values are in italics and marked with an asterisk

VD subsection group	Slab	ANOVA *p* value across all follow-up visits	ANOVA *p* value versus control	ANOVA *p* value across subsections
IN-IS-IT-II	Retina	> 0.0553	< 0.003*	> 0.4601
	SCP	> 0.0614	< 0.002*	> 0.1255
	DCP	> 0.0683	< 0.0001*	> 0.5482
ON-OS-OT-OI	Retina	> 0.0507	< 0.0005*	< 0.006*
	SCP	> 0.0967	< 0.0006*	< 0.001*
	DCP	> 0.1492	< 0.0001*	< 0.0468* (BL & 1W), else > 0.1892
All C1 subsections	Retina	< 0.0409* (C1IN & C1ST), else > 0.1090	< 0.009*	< 0.001*
	SCP	> 0.0578	< 0.005*	< 0.001*
	DCP	> 0.0654	< 0.0001*	< 0.001*
All C2 subsections	Retina	0.0380* (C2TS), else > 0.0924	< 0.03*	< 0.001*
	SCP	0.0152* (C2TS), else > 0.1020	< 0.0002*	< 0.001*
	DCP	> 0.0834	< 0.0001*	< 0.0035*
All C3 subsections	Retina	> 0.0962	0.0034* (C3NS), else > 0.08	< 0.011* (BL & TS), else > 0.0785
	SCP	> 0.0858	< 0.006*	< 0.0011*
	DCP	0.0368* (C3NI), else > 0.1964	< 0.01*	> 0.5143

**Table 3:** Results of the perfusion density (PD) analysis for all retinal OCTA slabs (retina, SCP, and DCP) are presented as *p* values (ANOVA) between all four follow-up visits (BL, TS, 1W, 6M) and all subsections as indicated with the extended ETDRS grid in Fig. 1. Significant *p* values are in italics and marked with an asterisk

PD subsection group	Slab	ANOVA *p* value across all follow-up visits	ANOVA *p* value versus control	ANOVA *p* value across subsections
IN-IS-IT-II	Retina	> 0.0690	< 0.02*	> 0.3337
	SCP	> 0.1032	< 0.0005*	> 0.1240
	DCP	> 0.0623	< 0.0001*	> 0.5765
ON-OS-OT-OI	Retina	> 0.0774	< 0.006*	< 0.0126*
	SCP	> 0.1296	< 0.003*	< 0.001*
	DCP	> 0.2078	< 0.0001*	0.0130* (BL), else > 0.0558
All C1 subsections	Retina	0.0441* (C1IN), else > 0.0658	< 0.05*	< 0.001*
	SCP	> 0.0830	< 0.0003*	< 0.001*
	DCP	> 0.0994	< 0.0001*	< 0.001*
All C2 subsections	Retina	> 0.0553	< 0.04*	< 0.001*
	SCP	0.0288* (C2TS), else > 0.1484	< 0.0001*	< 0.001*
	DCP	> 0.1073	< 0.0001*	< 0.0049*
All C3 subsections	Retina	> 0.0874	0.004* (C3NS), else > 0.07	< 0.488* (BL, TS, 1 W), else 0.3346
	SCP	> 0.1068	< 0.03*	< 0.0017*
	DCP	0.0417* (C3NI), else > 0.2135	< 0.009*	> 0.5515

Furthermore, the average PD was measured 0.32 ± 0.06 at BL, 0.28 ± 0.10 at TS (*p* = 0.14), 0.28 ± 0.09 at 1W after stimulation (*p* = 0.07), and 0.27 ± 0.08 at the 6M follow-up (*p* = 0.054, Table 1, Table 3, Fig. 3). When analysed separately in the SCP and DCP, similar findings were observed (Fig. 3).

When the VD and PD were analysed over all subsections throughout the course of TES treatment, only few pairwise comparisons revealed significant differences: the VD in the C1ST subsection of the C1 ring (see Fig. 1) in the retinal slab revealed a significant decrease from 24.06 ± 7.55 at BL to 18.33 ± 9.54 mm/mm^2^ at 1W (*p* = 0.0265, Table 2, Supplementary Table S1C). Also, the VD in the C2TS subsection of the C2 ring for both the retinal and SCP slab presented with significant decrements from 18.20 ± 7.59 at BL to 12.86 ± 8.85 mm/mm^2^ at 1W and from 12.66 ± 7.20 at BL to 7.55 ± 6.04 mm/mm^2^ at 1W (*p* = 0.0326 and *p* = 0.0069, Table 2, Supplementary Table S1D). Also, for the PD, only few significant changes could be detected: the C1ST subsection of the C1 ring (see Fig. 1) in the retinal slab revealed a significant decrease from 0.41 ± 0.09 at BL to 0.33 ± 0.13 at 6M (*p* = 0.0465, Supplementary Table S2C). Similar to VD, also the PD in the C2TS subsection of the C2 ring in the SCP slab showed a significant decrease from 0.16 ± 0.09 at BL to 0.10 ± 0.08 at 1W (*p* = 0.0159, Table 3, Supplementary Table S2D). Furthermore, the C3NI subsection in the DCP slab of the C3 ring presented with a significant decrease of the PD from 0.18 ± 0.09 at BL to 0.15 ± 0.09 at 1W (*p* = 0.0206, Supplementary Table S2D). However, regarding the number of pairwise comparisons that were performed, these few differences should not be over interpreted. Therefore, in summary, the macular density metrics VD and PD seem to remain unaffected despite ongoing TES treatment.

#### Comparison of macular VD and PD in all subsections of the ETDRS grid

Independent from TES treatment, we found various differences for VD and PD between the different subsections within the extended ETDRS grid (Fig. 1). When analysed systematically within all subsections of all retinal slabs (retina, SCP, DCP), we found significant differences in the outer, C1, C2, and C3 rings. However, not within the inner ring (*p* > 0.87).

In the outer ring, the VD and PD presented with significantly lower values in the OT (outer temporal) subsection when compared with the other OS, ON, and OI subsections (*p* < 0.05 for VD and < 0.04 for PD, Supplementary Table S3A, B).

Also, in the peripheral C1, C2, and C3 rings, mainly the VD and PD of the temporal subsections (C1TS and C1 TI in the C1 ring, C2TS and C2TI in the C2 ring, and C3TS and C3TI in the C3 ring) were significantly lower when compared with the superior, nasal, and inferior subsections (*p* < 0.05, Supplementary Table S3A, B).

### Peripapillary microvasculature metrics

#### Comparison of peripapillary CPD and CFI at baseline, after first stimulation, 1 week, and 6 months after TES and versus control

When compared with healthy control eyes, all CPD and CFI values throughout the time course of TES therapy showed significantly lower values (*p* < 0.0001, Table 4). However, within RP eyes, we found only slight differences over the time course without reaching statistically significant values when the peripapillary nerve fibre layer microvasculature density parameters CPD and CFI were taken into account (Fig. 3, Tables 1 and 4). The temporal and superior CFI showed significant differences (*p* < 0.047) over all follow-up visits; however, when analysed further in pairwise comparisons, these changes were significant (*p* > 0.97) neither for these two subsections nor for the other subsections between all follow-up visits (*p* > 0.99, Table 1).

**Table 4:** Results of peripapillary microvasculature analysis such as the capillary perfusion density (CPD) and the capillary flux index (CFI) of the peripapillary nerve fibre layer microvasculature in eyes of patients suffering from retinitis pigmentosa undergoing TES treatment as mean ± standard deviation (SD) for all subsections of the grid shown in Fig 2 and at all follow-up visits (BL = baseline, TS = threshold, 1W = 1 week, and 6M = 6 months)

Follow-up	CFI average	CFI temporal	CFI superior	CFI nasal	CFI inferior	*p* value ANOVA subsections (T, S, N, I)
Control	0.36 ± 0.03	0.35 ± 0.03	0.37 ± 0.04	0.36 ± 0.04	0.35 ± 0.03	0.6708
BL	0.22 ± 0.06	0.22 ± 0.06	0.22 ± 0.06	0.21 ± 0.06	0.22 ± 0.05	0.9640
TS	0.18 ± 0.06	0.18 ± 0.07	0.18 ± 0.07	0.17 ± 0.06	0.18 ± 0.06	0.9139
1W	0.17 ± 0.06	0.17 ± 0.07	0.17 ± 0.07	0.18 ± 0.06	0.18 ± 0.06	0.9092
6M	0.23 ± 0.05	0.23 ± 0.06	0.24 ± 0.05	0.21 ± 0.04	0.22 ± 0.04	0.6618
ANOVA *p* value all visits (BL, TS, 1W, 6M)	0.0574	0.0460* (pairwise comparisons *p* > 0.97)	0.0348* (pairwise comparisons *p* > 0.92)	0.1353	0.1325	
ANOVA *p* value vs. control	< 0.0001*	< 0.0001*	< 0.0001*	< 0.0001*	< 0.0001*	
Follow-up	CPD average	CPD temporal	CPD superior	CPD nasal	CPD inferior	*p* value ANOVA subsections (T, S, N, I)
Control	0.57 ± 0.02	0.60 ± 0.02	0.56 ± 0.02	0.54 ± 0.04	0.57 ± 0.03	0.4703
BL	0.50 ± 0.05	0.53 ± 0.07	0.47 ± 0.04	0.49 ± 0.05	0.49 ± 0.05	0.0100*
TS	0.47 ± 0.04	0.50 ± 0.07	0.47 ± 0.04	0.45 ± 0.04	0.47 ± 0.05	0.0668
1W	0.49 ± 0.04	0.50 ± 0.06	0.48 ± 0.04	0.49 ± 0.05	0.49 ± 0.04	0.8313
6M	0.50 ± 0.03	0.54 ± 0.05	0.49 ± 0.03	0.48 ± 0.05	0.50 ± 0.04	0.0293*
ANOVA *p* value all visits (BL, TS, 1W, 6M)	0.3056	0.2561	0.4368	0.1524	0.3620	
ANOVA *p* value vs. control	< 0.0001*	< 0.0001*	< 0.0001*	< 0.0001*	< 0.0001*	

Significant *p* values are marked with an asterisk

#### Comparison of peripapillary CPD and CFI in all subsections of the segmentation ring

When comparing the CFI and CPD within the different subsections of the peripapillary ring (see Fig. 2), we found no significant pairwise comparison for the CFI (Supplementary Table S3C). However, for the CPD, the temporal subsection showed significant differences when compared with the superior section at the BL follow-up (*p* = 0.0054) and when compared with the nasal section both at the TS (*p* = 0.0436) and 6M follow-up (*p* = 0.0310, Supplementary Table S3C).

## Discussion

With few exceptions such as gene therapy for RPE65-associated retinal dystrophy, TES is currently the only evidence-based and clinically available method to slow down disease progression in RP [1, 2]. A recent study suggested retinal vessel oximetry to possibly serve as a sensitive monitoring method for TES therapy [3]. However, since these devices are preserved to specialised ophthalmology centres, we aimed to investigate whether OCTA might also show changes that are attributed to TES treatment.

Our results indicate that vascular density metrics in the macular region and the peripapillary microvasculature remain unaffected despite ongoing TES treatment, at least in a 6-month interval. There are several explanations for our findings: the mutated genes expressed in the retinal photoreceptors or retinal pigment epithelium [31] in RP induce apoptosis of the involved sensory retina [32]. With progression of atrophy, the neural retina responds with secondary remodelling such as proximal intraretinal pigment migration followed by inner retinal atrophy, neuronal or glial migration, and neurovascular remodelling [32]. These structural changes seem to be not as dynamic as the retinal vessel oxygen metabolism [3]. Furthermore, our findings are compatible with preliminary data showing that TES does not have a significant effect on the diameter of large peripapillary vessels [3]. Our study confirms that also the microvascular changes in the different retinal layers seem to follow the same mechanism and thus remain unaffected despite ongoing TES treatment. One further possible explanation might be the maximal follow-up of 6 months. Since neurovascular remodelling in RP seems not to change significantly over a period of 6 months, further studies with long-term outcomes of TES on OCTA parameters are needed to provide a clear evidence-based recommendation for or against the usage of OCTA for TES therapy monitoring.

Several studies have focused on the microvascular changes in RP using OCTA [13–27]. However, to the best of our knowledge, this is the first study reporting on longitudinal observations in RP. Furthermore, publications reporting on microvascular changes in RP were mainly presenting mean values in smaller parafoveal scans [15, 18–20, 25]. By using wide-field swept-source OCTA, a systematical analysis within all subsections of a standardised extended ETDRS grid could be performed and revealed significant differences in the vascular density metrics of the macular region.

In particular, we found the VD and PD values of the temporal subsections in the outer as well as in the peripheral C1, C2, and C3 rings to be significantly lower than the other subsections, while the inner ring did not present with significant differences. Our results are in accordance with current OCTA studies: Mastropasqua et al. investigated on the PD and vessel length density in 12 RP patients by using three rings of different sizes centred around the fovea and found a significant reduction of the PD and vessel length density in the central and peripheral retinal areas of RP patients when compared with healthy subjects [12]. Koyanagi et al. investigated on microvascular changes in RP by using a foveal and parafoveal ring within small OCTA scans of 3 × 3 mm and found the foveal and parafoveal flow densities to be significantly reduced when compared with controls [21]. Also, Toto et al. performed VD analyses by using a foveal and parafoveal ring and found the parafoveal rather than the foveal regions to present with significant reductions in comparison with controls [24].

In healthy subjects, the mean VDs were not reported to show significant differences within quadrants: in a large study with 1631 participants, You et al. found the mean superficial VD measured within a foveal and parafoveal ring to be slightly higher in the superior quadrant followed by the temporal, nasal, and inferior quadrants. In the deep retinal layer, VD was also found to be the highest in the superior quadrant followed by nasal, inferior, and temporal quadrants. However, the authors did not report any significant differences between the subsections [33]. Moreover, our findings of reduced vascular density metrics especially in the temporal subsections of the retina are in accordance with current investigations on the metabolic dysfunction in RP. Bojinova et al. found a nasal–temporal difference in the oxygen metabolism within rod–cone dystrophies such as RP [9]. They explained these findings with the variations in human photoreceptors’ topography: in the peripheral nasal retina relative to the temporal retina, a higher cone density was found [34]. On the contrary, rods seem to have a higher density in the peripheral temporal areas [34]. Since rod–cone dystrophies such as classic RP present mainly with impaired rod function, it is to be expected to find signs of degeneration also in OCTA with significant changes of the microvascular structures especially in the temporal regions as we could now confirm in our present study.

Furthermore, when performing OCTA studies in RP, the examined groups are often mixed with and without the presence of macular oedema [15, 18–20, 22, 23, 25–27]. However, as described previously, macular oedema has a significant impact on OCTA parameters [11]. This might explain why some studies presented contradictory results, e.g. with larger foveal avascular zones in RP when compared with controls, while others reported smaller zones [19] or found no significant difference at all [21]. Furthermore, parameters such as avascular zones, especially in the DCP layer, are manual measurements and therefore not very reliable. Hence, we only included RP patients without macular oedema in this study and used solely parameters from fully automated measurements.

Due to its exploratory nature, our study has a number of limitations: a small sample size, a relatively short follow-up of 6 months, the genetic heterogeneity of RP patients, and the lack of a sham-controlled RP group.

In conclusion, the microvascular changes in RP measured with OCTA appear to remain unaffected by ongoing TES treatment. However, our wide-field swept-source OCTA approach with a systematic analysis of all subsections in a standardised extended ETDRS grid revealed significant changes of the microvascular structures especially in the temporal retinal areas in eyes suffering from RP.

## Electronic supplementary material

Table S1 (DOCX 31 kb)

Table S2 (DOCX 29 kb)

Table S3 (DOCX 37 kb)
