# Supplementary material for: Optical coherence tomography angiography findings in patients undergoing transcorneal electrical stimulation for treating retinitis pigmentosa
Source: Graefes Arch Clin Exp Ophthalmol. 2020 Oct 10;259(5):1167–77. doi: 10.1007/s00417-020-04963-7 (PMC8102288; doi:10.1007/s00417-020-04963-7)
Supplement: Supplementary file 1 — (DOCX 31 kb) [file 417_2020_4963_MOESM1_ESM.docx]

| **slab** | **follow-up** | **average** | **C** | **Inner Ring** | **IN** | **IS** | **IT** | **II** | **p-value ANOVA subsections (IN, IS, IT, II)** |
| --- | --- | --- | --- | --- | --- | --- | --- | --- | --- |
| **Retina** | **BL** | 24.13 ± 4.71 | 15.95 ± 8.73 | 26.35 ± 8.74 | 26.70 ± 9.36 | 27.05 ± 9.92 | 24.34 ± 9.26 | 27.29 ± 8.81 | 0.4601 |
| **Retina** | **TS** | 21.15 ± 7.09 | 14.36 ± 9.08 | 23.33 ± 9.93 | 21.86 ± 11.03 | 23.98 ± 10.94 | 22.53 ± 10.16 | 24.93 ± 9.92 | 0.5445 |
| **Retina** | **1W** | 20.71 ± 6.96 | 13.71 ± 9.51 | 22.53 ± 9.93 | 21.69 ± 10.50 | 23.10 ± 10.67 | 20.80 ± 10.59 | 24.50 ± 10.22 | 0.4632 |
| **Retina** | **6M** | 20.90 ± 5.88 | 14.67 ± 8.13 | 22.67 ± 9.94 | 22.65 ± 10.66 | 24.26 ± 9.46 | 21.22 ± 10.18 | 22.53 ± 11.92 | 0.7822 |
| **p-value ANOVA** | **all visits** | 0.0553 | 0.7264 | 0.2693 | 0.1092 | 0.3611 | 0.4235 | 0.2926 |  |
| **SCP** | **BL** | 19.60 ± 4.55 | 15.22 ± 8.86 | 23.14 ± 8.41 | 23.99 ± 9.39 | 23.62 ± 9.36 | 20.74 ± 8.19 | 24.20 ± 8.93 | 0.2661 |
| **SCP** | **TS** | 17.07 ± 6.67 | 14.36 ± 9.31 | 20.64 ± 9.04 | 20.35 ± 10.58 | 21.39 ± 9.54 | 19.03 ± 9.01 | 21.76 ± 9.73 | 0.5855 |
| **SCP** | **1W** | 16.53 ± 5.93 | 12.94 ± 8.08 | 19.97 ± 8.25 | 19.90 ± 9.45 | 20.63 ± 8.63 | 17.16 ± 9.07 | 22.13 ± 8.68 | 0.1255 |
| **SCP** | **6M** | 16.57 ± 5.36 | 12.77 ± 7.02 | 18.94 ± 8.64 | 19.98 ± 9.40 | 19.90 ± 8.43 | 16.48 ± 8.25 | 19.36 ± 10.34 | 0.4699 |
| **p-value ANOVA** | **all visits** | 0.0614 | 0.5721 | 0.2078 | 0.1992 | 0.3426 | 0.1674 | 0.2319 |  |
| **DCP** | **BL** | 13.94 ± 5.11 | 8.01 ± 6.75 | 17.63 ± 8.39 | 17.61 ± 9.16 | 18.58 ± 8.96 | 16.04 ± 7.91 | 18.28 ± 8.83 | 0.5570 |
| **DCP** | **TS** | 12.29 ± 6.87 | 7.34 ± 7.85 | 15.00 ± 9.76 | 13.59 ± 9.89 | 16.05 ± 10.82 | 14.12 ± 9.28 | 16.20 ± 10.40 | 0.5482 |
| **DCP** | **1W** | 11.67 ± 5.28 | 7.38 ± 7.36 | 14.13 ± 8.28 | 13.38 ± 8.62 | 14.72 ± 9.35 | 13.42 ± 7.65 | 14.97 ± 8.99 | 0.7993 |
| **DCP** | **6M** | 10.93 ± 5.28 | 5.36 ± 5.08 | 12.59 ± 8.33 | 12.31 ± 8.16 | 13.40 ± 8.60 | 11.53 ± 7.96 | 13.12 ± 10.19 | 0.8674 |
| **p-value ANOVA** | **all visits** | 0.1589 | 0.4946 | 0.1172 | 0.0683 | 0.1408 | 0.1779 | 0.1703 |  |

**Supplementary Table S1A**: Results of the vessel density (VD) are presented as mean ± SD in mm/mm² for all retinal OCTA slabs (Retina, SCP and DCP) with corresponding p-values (ANOVA) between all four follow-up visits (BL, TS, 1W, 6M, vertical) and all subsections (IN, IS, IT, II, horizontal) of the inner ring as indicated with the extended ETDRS grid in Figure 1. Significant p-values are bold and marked with an asterisk.

| **slab** | **follow-up** | **Outer Ring** | **ON** | **OS** | **OT** | **OI** | **p-value ANOVA subsections (ON, OS, OT, OI)** |
| --- | --- | --- | --- | --- | --- | --- | --- |
| **Retina** | **BL** | 26.90 ± 6.12 | 30.94 ± 7.27 | 27.03 ± 6.64 | 20.87 ± 7.36 | 28.75 ± 6.26 | **<0.001 *** |
| **Retina** | **TS** | 23.14 ± 8.69 | 26.25 ± 10.66 | 22.85 ± 9.97 | 18.10 ± 9.07 | 25.34 ± 9.62 | **0.0011 *** |
| **Retina** | **1W** | 22.46 ± 8.46 | 26.57 ± 10.23 | 21.52 ± 9.59 | 16.77 ± 8.75 | 24.97 ± 9.64 | **<0.001 *** |
| **Retina** | **6M** | 23.10 ± 8.33 | 27.76 ± 9.18 | 23.31 ± 9.23 | 18.12 ± 8.00 | 23.19 ± 11.59 | **0.006 *** |
| **p-value ANOVA** | **all visits** | 0.0652 | 0.1168 | 0.0507 | 0.1901 | 0.0971 |  |
| **SCP** | **BL** | 24.33 ± 6.17 | 30.02 ± 7.81 | 23.88 ± 7.08 | 17.43 ± 6.01 | 25.97 ± 7.57 | **<0.001 *** |
| **SCP** | **TS** | 21.30 ± 8.31 | 25.75 ± 10.10 | 20.86 ± 9.89 | 15.62 ± 8.35 | 22.98 ± 9.81 | **<0.001 *** |
| **SCP** | **1W** | 20.47 ± 7.45 | 26.56 ± 9.83 | 19.59 ± 8.67 | 13.94 ± 7.31 | 21.77 ± 9.00 | **<0.001 *** |
| **SCP** | **6M** | 20.52 ± 8.19 | 26.73 ± 8.92 | 20.34 ± 9.19 | 13.81 ± 6.64 | 21.19 ± 10.88 | **<0.001 *** |
| **p-value ANOVA** | **all visits** | 0.0967 | 0.1861 | 0.1644 | 0.1225 | 0.1386 |  |
| **DCP** | **BL** | 15.36 ± 6.62 | 17.53 ± 7.67 | 15.77 ± 6.45 | 12.18 ± 7.29 | 15.95 ± 7.59 | **0.0119 *** |
| **DCP** | **TS** | 13.24 ± 8.45 | 14.20 ± 9.42 | 13.89 ± 8.95 | 10.71 ± 8.35 | 14.15 ± 9.23 | 0.2315 |
| **DCP** | **1W** | 12.27 ± 6.93 | 14.16 ± 8.36 | 12.08 ± 7.61 | 9.34 ± 6.95 | 13.48 ± 8.04 | **0.0468 *** |
| **DCP** | **6M** | 11.69 ± 7.47 | 14.03 ± 8.18 | 12.13 ± 7.79 | 9.15 ± 6.87 | 11.45 ± 9.30 | 0.1892 |
| **p-value ANOVA** | **all visits** | 0.1821 | 0.1492 | 0.2953 | 0.2164 | 0.2798 |  |

**Supplementary Table S1B**: Results of the vessel density (VD) are presented as mean ± SD in mm/mm² for all retinal OCTA slabs (Retina. SCP and DCP) with corresponding p-values (ANOVA) between all four follow-up visits (BL, TS, 1W, 6M, vertical) and all subsections (ON, OS, OT, OI, horizontal) of the outer ring as indicated with the extended ETDRS grid in Figure 1. Significant p-values are bold and marked with an asterisk.

| **slab** | **follow-up** | **C1NS** | **C1NI** | **C1TS** | **C1TI** | **C1SN** | **C1IN** | **C1ST** | **C1IT** | **p-value ANOVA all C1 subsections** |
| --- | --- | --- | --- | --- | --- | --- | --- | --- | --- | --- |
| **Retina** | **BL** | 29.88 ± 7.04 | 34.34 ± 6.01 | 17.54 ± 7.27 | 17.99 ± 6.61 | 28.79 ± 7.67 | 31.18 ± 6.80 | 24.06 ± 7.55 | 25.05 ± 7.87 | **<0.001 *** |
| **Retina** | **TS** | 25.98 ± 9.38 | 30.97 ± 8.90 | 14.32 ± 9.24 | 16.91 ± 10.45 | 24.83 ± 10.40 | 26.71 ± 9.52 | 19.71 ± 9.70 | 21.01 ± 11.14 | **<0.001 *** |
| **Retina** | **1W** | 25.83 ± 10.59 | 31.20 ± 8.60 | 13.73 ± 8.68 | 15.11 ± 9.90 | 23.70 ± 11.10 | 26.44 ± 10.65 | 18.33 ± 9.54 | 21.84 ± 10.89 | **<0.001 *** |
| **Retina** | **6M** | 29.09 ± 7.58 | 30.33 ± 9.91 | 13.85 ± 8.16 | 14.98 ± 8.67 | 25.09 ± 9.37 | 25.16 ± 9.85 | 20.10 ± 8.25 | 20.43 ± 9.29 | **<0.001 *** |
| **p-value ANOVA** | **all visits** | 0.1090 | 0.1764 | 0.1655 | 0.4439 | 0.1227 | **0.0409 *** | **0.0357 ***  (0.0265 *  BL vs 1W) | 0.2104 |  |
| **SCP** | **BL** | 28.64 ± 7.59 | 33.61 ± 6.41 | 13.39 ± 6.23 | 15.06 ± 6.09 | 26.19 ± 8.70 | 27.67 ± 9.13 | 19.80 ± 8.61 | 21.58 ± 8.08 | **<0.001 *** |
| **SCP** | **TS** | 24.91 ± 9.38 | 30.05 ± 9.52 | 11.52 ± 7.95 | 14.19 ± 9.12 | 23.22 ± 10.94 | 23.62 ± 10.46 | 15.50 ± 9.80 | 17.84 ± 10.86 | **<0.001 *** |
| **SCP** | **1W** | 25.33 ± 10.86 | 30.92 ± 9.06 | 10.06 ± 6.78 | 11.45 ± 8.02 | 21.94 ± 11.25 | 24.08 ± 10.95 | 14.62 ± 9.21 | 18.03 ± 10.08 | **<0.001 *** |
| **SCP** | **6M** | 27.86 ± 7.21 | 29.66 ± 9.75 | 10.84 ± 6.46 | 11.81 ± 6.91 | 22.52 ± 9.97 | 22.61 ± 9.86 | 15.20 ± 8.43 | 17.72 ± 9.55 | **<0.001 *** |
| **p-value ANOVA** | **all visits** | 0.2002 | 0.2124 | 0.2033 | 0.1352 | 0.2927 | 0.1804 | 0.0578 | 0.2576 |  |
| **DCP** | **BL** | 19.28 ± 7.20 | 20.48 ± 7.16 | 9.54 ± 5.98 | 8.80 ± 5.42 | 16.89 ± 7.98 | 16.84 ± 7.66 | 14.10 ± 6.91 | 13.68 ± 7.65 | **<0.001 *** |
| **DCP** | **TS** | 15.92 ± 9.51 | 17.09 ± 9.35 | 7.98 ± 7.17 | 9.32 ± 8.21 | 14.95 ± 9.36 | 14.71 ± 9.76 | 12.23 ± 8.46 | 12.28 ± 9.63 | **<0.001 *** |
| **DCP** | **1W** | 16.01 ± 9.32 | 16.91 ± 8.87 | 6.99 ± 6.08 | 7.84 ± 7.12 | 13.51 ± 8.84 | 13.49 ± 8.42 | 11.00 ± 7.09 | 11.93 ± 8.89 | **<0.001 *** |
| **DCP** | **6M** | 16.66 ± 8.17 | 14.87 ± 8.92 | 7.81 ± 6.43 | 7.06 ± 7.05 | 13.35 ± 8.15 | 11.44 ± 7.27 | 10.43 ± 6.06 | 8.78 ± 7.28 | **<0.001 *** |
| **p-value ANOVA** | **all visits** | 0.0654 | 0.3845 | 0.5728 | 0.2870 | 0.0882 | 0.1688 | 0.1798 | 0.6805 |  |

**Supplementary Table S1C**: Results of the vessel density (VD) are presented as mean ± SD in mm/mm² for all retinal OCTA slabs (Retina. SCP and DCP) with corresponding p-values (ANOVA) between all four follow-up visits (BL, TS, 1W, 6M, vertical) and all subsections (C1NS, C1NI, C1TS, C1TI, C1SN, C1IN, C1ST, C1IT, horizontal) of the C1-ring as indicated with the extended ETDRS grid in Figure 1. Significant p-values are bold and marked with an asterisk. P-values in brackets indicate significant pairwise comparisons between two follow-up visits which are specified below.

| **slab** | **follow-up** | **C2NS** | **C2NI** | **C2TS** | **C2TI** | **C3NS** | **C3NI** | **C3TS** | **C3TI** | **p-value ANOVA all C2 subsections** | **p-value ANOVA all C3 subsections** |
| --- | --- | --- | --- | --- | --- | --- | --- | --- | --- | --- | --- |
| **Retina** | **BL** | 27.96 ± 6.81 | 30.63 ± 6.39 | 18.20 ± 7.59 | 19.27 ± 7.34 | 26.13 ± 7.05 | 25.71 ± 7.01 | 19.58 ± 8.57 | 21.38 ± 10.56 | **<0.001 *** | **0.0012 *** |
| **Retina** | **TS** | 24.77 ± 8.39 | 27.07 ± 9.00 | 13.89 ± 9.68 | 17.68 ± 10.31 | 21.47 ± 10.19 | 23.45 ± 10.50 | 16.16 ± 9.52 | 18.85 ± 9.89 | **<0.001 *** | **0.0111 *** |
| **Retina** | **1W** | 25.03 ± 8.98 | 27.30 ± 9.40 | 12.86 ± 8.85 | 17.00 ± 10.00 | 22.14 ± 10.70 | 22.43 ± 9.71 | 16.64 ± 10.45 | 19.66 ± 9.91 | **<0.001 *** | 0.0785 |
| **Retina** | **6M** | 26.25 ± 7.92 | 25.69 ± 8.42 | 14.50 ± 6.83 | 15.93 ± 7.63 | 21.32 ± 8.62 | 20.13 ± 9.33 | 16.34 ± 9.95 | 18.65 ± 11.14 | **<0.001 *** | 0.3223 |
| **p-value ANOVA** | **all visits** | 0.2948 | 0.0924 | **0.0380 ***  (0.0326 *  BL vs. 1W) | 0.4978 | 0.0962 | 0.1240 | 0.3831 | 0.6858 |  |  |
| **SCP** | **BL** | 25.66 ± 7.36 | 26.37 ± 7.78 | 12.66 ± 7.20 | 13.11 ± 6.46 | 20.59 ± 7.94 | 18.41 ± 7.17 | 11.93 ± 6.80 | 12.04 ± 7.17 | **<0.001 *** | **<0.001 *** |
| **SCP** | **TS** | 22.25 ± 8.85 | 22.89 ± 9.27 | 9.39 ± 7.44 | 12.48 ± 8.46 | 15.79 ± 9.74 | 16.13 ± 8.87 | 8.61 ± 6.80 | 10.36 ± 7.11 | **<0.001 *** | **<0.001 *** |
| **SCP** | **1W** | 23.30 ± 8.67 | 24.07 ± 8.92 | 7.55 ± 6.04 | 10.06 ± 7.04 | 17.58 ± 10.29 | 14.95 ± 9.16 | 9.09 ± 8.35 | 9.45 ± 6.18 | **<0.001 *** | **<0.001 *** |
| **SCP** | **6M** | 24.39 ± 7.23 | 21.96 ± 8.74 | 9.97 ± 6.11 | 9.47 ± 5.67 | 17.09 ± 8.83 | 13.16 ± 7.49 | 9.11 ± 6.60 | 9.64 ± 7.20 | **<0.001 *** | **0.0011 *** |
| **p-value ANOVA** | **all visits** | 0.2986 | 0.1778 | **0.0152 ***  (0.0069 *  BL vs. 1W) | 0.1020 | 0.1462 | 0.0858 | 0.1832 | 0.4044 |  |  |
| **DCP** | **BL** | 16.09 ± 6.83 | 15.59 ± 6.71 | 10.16 ± 7.69 | 9.96 ± 6.47 | 14.28 ± 7.21 | 14.62 ± 7.07 | 12.69 ± 8.66 | 14.34 ± 11.17 | **<0.001 *** | 0.7625 |
| **DCP** | **TS** | 14.42 ± 8.75 | 13.87 ± 8.04 | 8.19 ± 7.82 | 10.58 ± 8.74 | 12.19 ± 8.51 | 12.73 ± 8.14 | 10.20 ± 9.00 | 12.45 ± 9.12 | **0.0032 *** | 0.5748 |
| **DCP** | **1W** | 14.32 ± 7.85 | 13.23 ± 7.43 | 7.01 ± 5.43 | 9.52 ± 9.01 | 12.37 ± 7.56 | 12.11 ± 7.27 | 12.32 ± 9.28 | 12.94 ± 9.66 | **0.0002 *** | 0.9820 |
| **DCP** | **6M** | 14.13 ± 7.09 | 10.82 ± 6.83 | 8.49 ± 5.84 | 8.19 ± 5.60 | 10.07 ± 6.50 | 9.06 ± 6.42 | 11.30 ± 9.78 | 12.85 ± 12.63 | **0.0035 *** | 0.5143 |
| **p-value ANOVA** | **all visits** | 0.6914 | 0.0834 | 0.2689 | 0.6680 | 0.1964 | **0.0362 *** | 0.6458 | 0.8790 |  |  |

**Supplementary Table S1D**: Results of the vessel density (VD) are presented as mean ± SD in mm/mm² for all retinal OCTA slabs (Retina. SCP and DCP) with corresponding p-values (ANOVA) between all four follow-up visits (BL, TS, 1W, 6M, vertical) and all subsections (C2NS, C2NI, C2TS, C2TI or C3NS, C3NI, C3TS, C3TI, horizontal) of the C2 and C3-ring as indicated with the extended ETDRS grid in Figure 1. Significant p-values are bold and marked with an asterisk. P-values in brackets indicate significant pairwise comparisons between two follow-up visits which are specified below.
