# Supplementary material for: Optical coherence tomography angiography findings in patients undergoing transcorneal electrical stimulation for treating retinitis pigmentosa
Source: Graefes Arch Clin Exp Ophthalmol. 2020 Oct 10;259(5):1167–77. doi: 10.1007/s00417-020-04963-7 (PMC8102288; doi:10.1007/s00417-020-04963-7)
Supplement: Supplementary file 2 — (DOCX 29 kb) [file 417_2020_4963_MOESM2_ESM.docx]

| **slab** | **follow-up** | **average** | **C** | **Inner Ring** | **IN** | **IS** | **IT** | **II** | **p-value ANOVA subsections (IN, IS, IT, II)** |
| --- | --- | --- | --- | --- | --- | --- | --- | --- | --- |
| **Retina** | **BL** | 0.32 ± 0.06 | 0.20 ± 0.11 | 0.34 ± 0.11 | 0.34 ± 0.11 | 0.35 ± 0.12 | 0.32 ± 0.12 | 0.36 ± 0.11 | 0.4330 |
| **Retina** | **TS** | 0.28 ± 0.10 | 0.18 ± 0.12 | 0.30 ± 0.12 | 0.28 ± 0.14 | 0.31 ± 0.13 | 0.29 ± 0.13 | 0.33 ± 0.13 | 0.3926 |
| **Retina** | **1W** | 0.28 ± 0.09 | 0.18 ± 0.13 | 0.29 ± 0.12 | 0.28 ± 0.13 | 0.30 ± 0.13 | 0.27 ± 0.14 | 0.32 ± 0.13 | 0.3337 |
| **Retina** | **6M** | 0.27 ± 0.08 | 0.18 ± 0.10 | 0.28 ± 0.12 | 0.28 ± 0.13 | 0.30 ± 0.12 | 0.26 ± 0.12 | 0.29 ± 0.15 | 0.7644 |
| **p-value ANOVA** | **all visits** | **0.0309 *** | 0.7498 | 0.1474 | 0.0690 | 0.2443 | 0.2625 | 0.1389 |  |
| **SCP** | **BL** | 0.26 ± 0.06 | 0.19 ± 0.11 | 0.30 ± 0.10 | 0.30 ± 0.11 | 0.30 ± 0.11 | 0.27 ± 0.11 | 0.32 ± 0.11 | 0.2821 |
| **SCP** | **TS** | 0.22 ± 0.09 | 0.18 ± 0.11 | 0.27 ± 0.11 | 0.26 ± 0.13 | 0.28 ± 0.12 | 0.25 ± 0.12 | 0.29 ± 0.12 | 0.4932 |
| **SCP** | **1W** | 0.21 ± 0.08 | 0.16 ± 0.10 | 0.26 ± 0.10 | 0.25 ± 0.11 | 0.26 ± 0.10 | 0.23 ± 0.12 | 0.29 ± 0.11 | 0.1240 |
| **SCP** | **6M** | 0.21 ± 0.07 | 0.16 ± 0.09 | 0.24 ± 0.11 | 0.24 ± 0.11 | 0.25 ± 0.11 | 0.21 ± 0.10 | 0.24 ± 0.13 | 0.4988 |
| **p-value ANOVA** | **all visits** | 0.0442 | 0.4989 | 0.1002 | 0.1295 | 0.1927 | 0.1032 | 0.1081 |  |
| **DCP** | **BL** | 0.17 ± 0.06 | 0.10 ± 0.08 | 0.21 ± 0.10 | 0.21 ± 0.11 | 0.22 ± 0.10 | 0.20 ± 0.10 | 0.22 ± 0.10 | 0.6667 |
| **DCP** | **TS** | 0.15 ± 0.08 | 0.09 ± 0.09 | 0.18 ± 0.12 | 0.16 ± 0.12 | 0.19 ± 0.13 | 0.17 ± 0.11 | 0.19 ± 0.12 | 0.5765 |
| **DCP** | **1W** | 0.14 ± 0.06 | 0.09 ± 0.09 | 0.17 ± 0.10 | 0.16 ± 0.10 | 0.18 ± 0.11 | 0.17 ± 0.09 | 0.18 ± 0.11 | 0.7742 |
| **DCP** | **6M** | 0.13 ± 0.07 | 0.06 ± 0.06 | 0.15 ± 0.10 | 0.15 ± 0.10 | 0.16 ± 0.10 | 0.14 ± 0.09 | 0.15 ± 0.12 | 0.8908 |
| **p-value ANOVA** | **all visits** | 0.1251 | 0.4202 | 0.0855 | 0.0623 | 0.1152 | 0.1107 | 0.1234 |  |

**Supplementary Table S2A**: Results of the perfusion density (PD) are presented as mean ± SD for all retinal OCTA slabs (Retina, SCP and DCP) with corresponding p-values (ANOVA) between all four follow-up visits (BL, TS, 1W, 6M, vertical) and all subsections (IN, IS, IT, II, horizontal) of the inner ring as indicated with the extended ETDRS grid in Figure 1. Significant p-values are bold and marked with an asterisk.

| **slab** | **follow-up** | **Outer Ring** | **ON** | **OS** | **OT** | **OI** | **p-value ANOVA subsections (ON, OS, OT, OI)** |
| --- | --- | --- | --- | --- | --- | --- | --- |
| **Retina** | **BL** | 0.35 ± 0.08 | 0.39 ± 0.09 | 0.35 ± 0.09 | 0.27 ± 0.10 | 0.38 ± 0.08 | **<0.001 *** |
| **Retina** | **TS** | 0.31 ± 0.11 | 0.34 ± 0.13 | 0.31 ± 0.13 | 0.24 ± 0.12 | 0.34 ± 0.12 | **0.0016 *** |
| **Retina** | **1W** | 0.30 ± 0.11 | 0.34 ± 0.12 | 0.29 ± 0.12 | 0.23 ± 0.13 | 0.33 ± 0.12 | **<0.001 *** |
| **Retina** | **6M** | 0.30 ± 0.11 | 0.35 ± 0.12 | 0.30 ± 0.12 | 0.23 ± 0.10 | 0.30 ± 0.15 | **0.0126 *** |
| **p-value ANOVA** | **all visits** | 0.0863 | 0.1206 | 0.0774 | 0.3874 | 0.0804 |  |
| **SCP** | **BL** | 0.31 ± 0.08 | 0.37 ± 0.10 | 0.31 ± 0.10 | 0.23 ± 0.08 | 0.33 ± 0.10 | **<0.001 *** |
| **SCP** | **TS** | 0.28 ± 0.11 | 0.32 ± 0.13 | 0.27 ± 0.13 | 0.21 ± 0.12 | 0.30 ± 0.13 | **<0.001 *** |
| **SCP** | **1W** | 0.26 ± 0.09 | 0.33 ± 0.12 | 0.25 ± 0.11 | 0.19 ± 0.12 | 0.28 ± 0.11 | **<0.001 *** |
| **SCP** | **6M** | 0.26 ± 0.11 | 0.33 ± 0.11 | 0.26 ± 0.12 | 0.18 ± 0.09 | 0.27 ± 0.14 | **<0.001 *** |
| **p-value ANOVA** | **all visits** | 0.1141 | 0.1752 | 0.1904 | 0.2790 | 0.1296 |  |
| **DCP** | **BL** | 0.18 ± 0.08 | 0.21 ± 0.09 | 0.19 ± 0.08 | 0.15 ± 0.09 | 0.19 ± 0.09 | **0.0130 *** |
| **DCP** | **TS** | 0.16 ± 0.10 | 0.17 ± 0.11 | 0.17 ± 0.11 | 0.13 ± 0.10 | 0.17 ± 0.11 | 0.2165 |
| **DCP** | **1W** | 0.15 ± 0.08 | 0.17 ± 0.10 | 0.15 ± 0.09 | 0.12 ± 0.08 | 0.16 ± 0.10 | 0.0558 |
| **DCP** | **6M** | 0.14 ± 0.09 | 0.17 ± 0.10 | 0.15 ± 0.10 | 0.11 ± 0.08 | 0.14 ± 0.11 | 0.2066 |
| **p-value ANOVA** | **all visits** | 0.1968 | 0.2238 | 0.1904 | 0.3207 | 0.2078 |  |

**Supplementary Table S2B**: Results of the perfusion density (PD) are presented as mean ± SD for all retinal OCTA slabs (Retina, SCP and DCP) with corresponding p-values (ANOVA) between all four follow-up visits (BL, TS, 1W, 6M, vertical) and all subsections (ON, OS, OT, OI, horizontal) of the outer ring as indicated with the extended ETDRS grid in Figure 1. Significant p-values are bold and marked with an asterisk.

| **slab** | **follow-up** | **C1NS** | **C1NI** | **C1TS** | **C1TI** | **C1SN** | **C1IN** | **C1ST** | **C1IT** | **p-value ANOVA all C1 subsections** |
| --- | --- | --- | --- | --- | --- | --- | --- | --- | --- | --- |
| **Retina** | **BL** | 0.40 ± 0.09 | 0.45 ± 0.09 | 0.23 ± 0.09 | 0.24 ± 0.09 | 0.38 ± 0.10 | 0.41 ± 0.09 | 0.32 ± 0.10 | 0.33 ± 0.11 | **<0.001 *** |
| **Retina** | **TS** | 0.35 ± 0.12 | 0.41 ± 0.11 | 0.19 ± 0.13 | 0.23 ± 0.14 | 0.34 ± 0.14 | 0.36 ± 0.13 | 0.27 ± 0.14 | 0.29 ± 0.15 | **<0.001 *** |
| **Retina** | **1W** | 0.34 ± 0.13 | 0.41 ± 0.10 | 0.19 ± 0.13 | 0.21 ± 0.14 | 0.32 ± 0.14 | 0.35 ± 0.14 | 0.25 ± 0.13 | 0.29 ± 0.14 | **<0.001 *** |
| **Retina** | **6M** | 0.37 ± 0.10 | 0.39 ± 0.13 | 0.18 ± 0.11 | 0.19 ± 0.11 | 0.33 ± 0.13 | 0.33 ± 0.13 | 0.26 ± 0.11 | 0.27 ± 0.12 | **<0.001 *** |
| **p-value ANOVA** | **all visits** | 0.1265 | 0.1319 | 0.3840 | 0.5204 | 0.1570 | **0.0441 ***  (0.0465 *  BL vs. 6M) | 0.0658 | 0.2249 |  |
| **SCP** | **BL** | 0.37 ± 0.10 | 0.43 ± 0.09 | 0.17 ± 0.08 | 0.19 ± 0.08 | 0.33 ± 0.11 | 0.35 ± 0.12 | 0.25 ± 0.11 | 0.28 ± 0.10 | **<0.001 *** |
| **SCP** | **TS** | 0.32 ± 0.12 | 0.39 ± 0.12 | 0.16 ± 0.11 | 0.19 ± 0.12 | 0.30 ± 0.14 | 0.31 ± 0.14 | 0.20 ± 0.13 | 0.23 ± 0.15 | **<0.001 *** |
| **SCP** | **1W** | 0.32 ± 0.14 | 0.39 ± 0.11 | 0.14 ± 0.11 | 0.16 ± 0.12 | 0.28 ± 0.14 | 0.31 ± 0.14 | 0.19 ± 0.12 | 0.24 ± 0.13 | **<0.001 *** |
| **SCP** | **6M** | 0.35 ± 0.09 | 0.37 ± 0.13 | 0.14 ± 0.08 | 0.15 ± 0.09 | 0.28 ± 0.13 | 0.28 ± 0.13 | 0.19 ± 0.11 | 0.22 ± 0.12 | **<0.001 *** |
| **p-value ANOVA** | **all visits** | 0.2891 | 0.1707 | 0.5157 | 0.2358 | 0.3060 | 0.2037 | 0.0830 | 0.3400 |  |
| **DCP** | **BL** | 0.23 ± 0.09 | 0.25 ± 0.09 | 0.11 ± 0.07 | 0.11 ± 0.06 | 0.20 ± 0.10 | 0.20 ± 0.09 | 0.17 ± 0.09 | 0.17 ± 0.10 | **<0.001 *** |
| **DCP** | **TS** | 0.20 ± 0.12 | 0.21 ± 0.11 | 0.10 ± 0.09 | 0.11 ± 0.10 | 0.18 ± 0.11 | 0.18 ± 0.12 | 0.15 ± 0.11 | 0.15 ± 0.11 | **<0.001 *** |
| **DCP** | **1W** | 0.19 ± 0.11 | 0.20 ± 0.11 | 0.09 ± 0.07 | 0.10 ± 0.09 | 0.16 ± 0.11 | 0.17 ± 0.10 | 0.14 ± 0.09 | 0.15 ± 0.11 | **<0.001 *** |
| **DCP** | **6M** | 0.20 ± 0.10 | 0.18 ± 0.11 | 0.09 ± 0.08 | 0.09 ± 0.09 | 0.16 ± 0.10 | 0.14 ± 0.09 | 0.13 ± 0.07 | 0.11 ± 0.09 | **<0.001 *** |
| **p-value ANOVA** | **all visits** | 0.3756 | 0.0994 | 0.5220 | 0.5857 | 0.3191 | 0.0997 | 0.1861 | 0.1588 |  |

**Supplementary Table S2C**: Results of the perfusion density (PD) are presented as mean ± SD for all retinal OCTA slabs (Retina, SCP and DCP) with corresponding p-values (ANOVA) between all four follow-up visits (BL, TS, 1W, 6M, vertical) and all subsections (C1NS, C1NI, C1TS, C1TI, C1SN, C1IN, C1ST, C1IT, horizontal) of the C1-ring as indicated with the extended ETDRS grid in Figure 1. Significant p-values are bold and marked with an asterisk. P-values in brackets indicate significant pairwise comparisons between two follow-up visits which are specified below.

| **slab** | **follow-up** | **C2NS** | **C2NI** | **C2TS** | **C2TI** | **C3NS** | **C3NI** | **C3TS** | **C3TI** | **p-value ANOVA all C2 subsections** | **p-value ANOVA all C3 subsections** |
| --- | --- | --- | --- | --- | --- | --- | --- | --- | --- | --- | --- |
| **Retina** | **BL** | 0.37 ± 0.10 | 0.40 ± 0.09 | 0.23 ± 0.10 | 0.25 ± 0.10 | 0.34 ± 0.09 | 0.33 ± 0.09 | 0.25 ± 0.11 | 0.27 ± 0.14 | **<0.001 *** | **<0.001 *** |
| **Retina** | **TS** | 0.33 ± 0.12 | 0.35 ± 0.12 | 0.18 ± 0.12 | 0.23 ± 0.13 | 0.28 ± 0.14 | 0.30 ± 0.14 | 0.20 ± 0.12 | 0.24 ± 0.13 | **<0.001 *** | **0.0089 *** |
| **Retina** | **1W** | 0.33 ± 0.13 | 0.36 ± 0.13 | 0.17 ± 0.12 | 0.22 ± 0.13 | 0.29 ± 0.14 | 0.29 ± 0.13 | 0.21 ± 0.13 | 0.25 ± 0.13 | **<0.001 *** | **0.0488 *** |
| **Retina** | **6M** | 0.35 ± 0.11 | 0.33 ± 0.11 | 0.19 ± 0.09 | 0.20 ± 0.10 | 0.27 ± 0.11 | 0.25 ± 0.12 | 0.21 ± 0.13 | 0.24 ± 0.15 | **<0.001 *** | 0.3346 |
| **p-value ANOVA** | **all visits** | 0.3055 | 0.0879 | 0.0553 | 0.4466 | 0.0874 | 0.1128 | 0.3580 | 0.6440 |  |  |
| **SCP** | **BL** | 0.33 ± 0.10 | 0.34 ± 0.11 | 0.16 ± 0.09 | 0.17 ± 0.09 | 0.26 ± 0.10 | 0.23 ± 0.09 | 0.15 ± 0.08 | 0.15 ± 0.09 | **<0.001 *** | **<0.001 *** |
| **SCP** | **TS** | 0.29 ± 0.12 | 0.29 ± 0.13 | 0.12 ± 0.09 | 0.16 ± 0.11 | 0.20 ± 0.13 | 0.20 ± 0.11 | 0.11 ± 0.09 | 0.13 ± 0.09 | **<0.001 *** | **<0.001 *** |
| **SCP** | **1W** | 0.30 ± 0.12 | 0.31 ± 0.12 | 0.10 ± 0.08 | 0.13 ± 0.10 | 0.22 ± 0.13 | 0.19 ± 0.12 | 0.11 ± 0.10 | 0.12 ± 0.08 | **<0.001 *** | **<0.001 *** |
| **SCP** | **6M** | 0.31 ± 0.10 | 0.28 ± 0.12 | 0.13 ± 0.08 | 0.12 ± 0.07 | 0.21 ± 0.11 | 0.16 ± 0.09 | 0.11 ± 0.08 | 0.12 ± 0.09 | **<0.001 *** | **0.0017 *** |
| **p-value ANOVA** | **all visits** | 0.3919 | 0.1784 | **0.0288 ***  (0.0159 *  BL vs. 1W) | 0.1484 | 0.1632 | 0.1068 | 0.2008 | 0.3616 |  |  |
| **DCP** | **BL** | 0.19 ± 0.08 | 0.19 ± 0.08 | 0.12 ± 0.09 | 0.12 ± 0.08 | 0.17 ± 0.09 | 0.18 ± 0.09 | 0.15 ± 0.11 | 0.17 ± 0.14 | **<0.001 *** | 0.7277 |
| **DCP** | **TS** | 0.18 ± 0.11 | 0.17 ± 0.10 | 0.10 ± 0.10 | 0.13 ± 0.11 | 0.15 ± 0.11 | 0.15 ± 0.10 | 0.12 ± 0.11 | 0.15 ± 0.11 | **0.0036 *** | 0.5832 |
| **DCP** | **1W** | 0.17 ± 0.10 | 0.16 ± 0.09 | 0.08 ± 0.06 | 0.12 ± 0.11 | 0.15 ± 0.09 | 0.15 ± 0.09 | 0.15 ± 0.11 | 0.15 ± 0.12 | **<0.001 *** | 0.9875 |
| **DCP** | **6M** | 0.17 ± 0.09 | 0.13 ± 0.08 | 0.10 ± 0.07 | 0.10 ± 0.07 | 0.12 ± 0.08 | 0.11 ± 0.08 | 0.14 ± 0.12 | 0.15 ± 0.16 | **0.0049 *** | 0.5515 |
| **p-value ANOVA** | **all visits** | 0.7451 | 0.1073 | 0.3193 | 0.6853 | 0.2135 | **0.0417 ***  (0.0206 *  BL vs. 1W) | 0.7029 | 0.8603 |  |  |

**Supplementary Table S2D**: Results of the perfusion density (PD) are presented as mean ± SD for all retinal OCTA slabs (Retina, SCP and DCP) with corresponding p-values (ANOVA) between all four follow-up visits (BL, TS, 1W, 6M, vertical) and all subsections (C2NS, C2NI, C2TS, C2TI or C3NS, C3NI, C3TS, C3TI, horizontal) of the C2 and C3-ring as indicated with the extended ETDRS grid in Figure 1. Significant p-values are bold and marked with an asterisk. P-values in brackets indicate significant pairwise comparisons between two follow-up visits which are specified below.
