# Supplementary material for: Optical coherence tomography angiography findings in patients undergoing transcorneal electrical stimulation for treating retinitis pigmentosa
Source: Graefes Arch Clin Exp Ophthalmol. 2020 Oct 10;259(5):1167–77. doi: 10.1007/s00417-020-04963-7 (PMC8102288; doi:10.1007/s00417-020-04963-7)
Supplement: Supplementary file 3 — (DOCX 37 kb) [file 417_2020_4963_MOESM3_ESM.docx]

| **p-values of** |  | **VD Retina Inner Ring** | |  |  | **VD SCP Inner Ring** | |  |  | **VD DCP Inner Ring** | |  |  |
| --- | --- | --- | --- | --- | --- | --- | --- | --- | --- | --- | --- | --- | --- |
| **pairwise comparisons** | | **BL** | **TS** | **1W** | **6M** | **BL** | **TS** | **1W** | **6M** | **BL** | **TS** | **1W** | **6M** |
| **IN** | **IS** | 0.9983 | 0.7977 | 0.9409 | 0.9466 | 0.9976 | 0.9626 | 0.9856 | 1.0000 | 0.9578 | 0.6879 | 0.915359 | 0.9696824 |
| **IN** | **IT** | 0.6627 | 0.9919 | 0.9841 | 0.9619 | 0.3553 | 0.9282 | 0.5649 | 0.5168 | 0.8478 | 0.9952 | 0.9999983 | 0.988586 |
| **IN** | **II** | 0.9919 | 0.5499 | 0.6673 | 1.0000 | 0.9996 | 0.9130 | 0.7165 | 0.9950 | 0.9852 | 0.6472 | 0.8658483 | 0.9871434 |
| **IS** | **IT** | 0.5544 | 0.9233 | 0.7889 | 0.7291 | 0.4673 | 0.6916 | 0.3538 | 0.5361 | 0.5502 | 0.8232 | 0.9210825 | 0.8681872 |
| **IS** | **II** | 0.9995 | 0.9773 | 0.9424 | 0.9344 | 0.9910 | 0.9982 | 0.8938 | 0.9966 | 0.9987 | 0.9999 | 0.9993153 | 0.9994452 |
| **IT** | **II** | 0.4813 | 0.7293 | 0.4405 | 0.9706 | 0.2991 | 0.5823 | 0.0865 | 0.6690 | 0.6492 | 0.7890 | 0.8731601 | 0.9140554 |
| **p-values of** |  | **VD Retina Outer Ring** | |  |  | **VD SCP Outer Ring** | |  |  | **VD DCP Outer Ring** | |  |  |
| **pairwise comparisons** | | **BL** | **TS** | **1W** | **6M** | **BL** | **TS** | **1W** | **6M** | **BL** | **TS** | **1W** | **6M** |
| **ON** | **OS** | 0.0596 | 0.4003 | 0.1129 | 0.3426 | 0.0009 | 0.0944 | 0.0041 | 0.0586 | 0.7083 | 0.9987 | 0.6672 | 0.8311 |
| **ON** | **OT** | **<0.001** | **0.0010** | **0.0001** | **0.0025** | **<0.001** | **<0.001** | **<0.001** | **<0.001** | **0.0063** | 0.2948 | **0.0419** | 0.1357 |
| **ON** | **OI** | 0.4997 | 0.9753 | 0.8935 | 0.3183 | 0.0591 | 0.5550 | 0.0932 | 0.1276 | 0.7702 | 1.0000 | 0.9827 | 0.6577 |
| **OS** | **OT** | **0.0005** | 0.1282 | 0.1498 | 0.2136 | **0.0004** | 0.0628 | **0.0312** | 0.0509 | 0.1281 | 0.3784 | 0.4383 | 0.5447 |
| **OS** | **OI** | 0.6878 | 0.6625 | 0.4197 | 1.0000 | 0.5696 | 0.7474 | 0.7157 | 0.9865 | 0.9996 | 0.9992 | 0.8700 | 0.9901 |
| **OT** | **OI** | **<0.001** | **0.0049** | 0.0016 | 0.2326 | **<0.001** | **0.0028** | **0.0008** | 0.0206 | 0.1008 | 0.3085 | 0.1065 | 0.7336 |
| **p-values of** |  | **VD Retina C1-ring** | |  |  | **VD SCP C1-ring** | |  |  | **VD DCP C1-ring** | |  |  |
| **pairwise comparisons** | | **BL** | **TS** | **1W** | **6M** | **BL** | **TS** | **1W** | **6M** | **BL** | **TS** | **1W** | **6M** |
| **C1NS** | **C1NI** | 0.0938 | 0.2670 | 0.2757 | 0.9996 | 0.0689 | 0.2200 | 0.1757 | 0.9939 | 0.9946 | 0.9989 | 0.9998 | 0.9880 |
| **C1NS** | **C1TS** | **<0.001** | **<0.001** | **<0.001** | **<0.001** | **<0.001** | **<0.001** | **<0.001** | **<0.001** | **<0.001** | **0.0013** | **<0.001** | **0.0004** |
| **C1NS** | **C1TI** | **<0.001** | **0.0005** | **0.0001** | **<0.001** | **<0.001** | **<0.001** | **<0.001** | **<0.001** | **<0.001** | **0.0177** | **0.0004** | **0.0001** |
| **C1NS** | **C1SN** | 0.9922 | 1.0000 | 1.0000 | 0.7553 | 0.9992 | 0.9988 | 0.9992 | 0.3214 | 0.7699 | 0.9988 | 0.8851 | 0.1885 |
| **C1NS** | **C1IN** | **0.0060** | 0.0632 | **0.0251** | **0.0051** | **<0.001** | **0.0002** | **<0.001** | **<0.001** | **0.0201** | 0.5662 | 0.1377 | **0.0454** |
| **C1NS** | **C1ST** | **0.0485** | 0.2823 | 0.6649 | **0.0108** | **0.0009** | **0.0192** | **0.0196** | **0.0004** | **0.0079** | 0.5895 | 0.3777 | **0.0037** |
| **C1NS** | **C1IT** | 0.9291 | 0.9993 | 1.0000 | 0.9392 | 0.6504 | 0.9178 | 0.9851 | 0.7978 | 0.4502 | 0.9953 | 0.9875 | 0.9193 |
| **C1NI** | **C1TS** | **<0.001** | **<0.001** | **<0.001** | **<0.001** | **<0.001** | **<0.001** | **<0.001** | **<0.001** | **<0.001** | **0.0001** | **<0.001** | **0.0122** |
| **C1NI** | **C1TI** | **<0.001** | **<0.001** | **<0.001** | **<0.001** | **<0.001** | **<0.001** | **<0.001** | **<0.001** | **<0.001** | **0.0019** | **<0.001** | **0.0031** |
| **C1NI** | **C1SN** | 0.4910 | 0.4885 | 0.4345 | 0.4185 | **0.0113** | **0.0487** | **0.0386** | 0.0517 | 0.2735 | 0.9306 | 0.6114 | 0.7164 |
| **C1NI** | **C1IN** | **<0.001** | **<0.001** | **<0.001** | **0.0006** | **<0.001** | **<0.001** | **<0.001** | **<0.001** | **0.0011** | 0.2055 | **0.0374** | 0.3610 |
| **C1NI** | **C1ST** | **<0.001** | **0.0001** | **0.0012** | **0.0016** | **<0.001** | **<0.001** | **<0.001** | **<0.001** | **0.0003** | 0.2224 | 0.1437 | 0.0660 |
| **C1NI** | **C1IT** | **0.0015** | 0.0764 | 0.1405 | 0.6957 | **0.0001** | **0.0061** | **0.0144** | 0.2919 | 0.0910 | 0.8816 | 0.8781 | 1.0000 |
| **C1TS** | **C1TI** | 1.0000 | 0.9268 | 0.9988 | 0.9998 | 0.9769 | 0.9108 | 0.9985 | 0.9999 | 0.9998 | 0.9974 | 0.9998 | 1.0000 |
| **C1TS** | **C1SN** | **<0.001** | **<0.001** | **<0.001** | **0.0001** | **<0.001** | **<0.001** | **<0.001** | **<0.001** | **0.0001** | **0.0150** | **0.0138** | 0.6575 |
| **C1TS** | **C1IN** | **0.0011** | 0.1824 | 0.4813 | 0.1617 | **0.0041** | 0.5564 | 0.4291 | 0.5442 | 0.0683 | 0.3709 | 0.4020 | 0.9045 |
| **C1TS** | **C1ST** | **0.0001** | **0.0376** | **0.0102** | 0.1340 | **<0.001** | 0.0567 | **0.0069** | 0.0631 | 0.1377 | 0.3656 | 0.1508 | 0.9998 |
| **C1TS** | **C1IT** | **<0.001** | **<0.001** | **<0.001** | **<0.001** | **<0.001** | **<0.001** | **<0.001** | **<0.001** | **0.0007** | **0.0262** | **0.0034** | **0.0399** |
| **C1TI** | **C1SN** | **<0.001** | **0.0001** | **<0.001** | **0.0010** | **<0.001** | **0.0002** | **<0.001** | **0.0001** | **<0.001** | 0.1136 | 0.0570 | 0.4086 |
| **C1TI** | **C1IN** | **0.0034** | 0.8944 | 0.8588 | 0.4033 | 0.0995 | 0.9986 | 0.8349 | 0.8152 | **0.0158** | 0.8166 | 0.7067 | 0.7148 |
| **C1TI** | **C1ST** | **0.0002** | 0.5401 | 0.0689 | 0.3477 | **0.0032** | 0.6690 | 0.0546 | 0.1806 | **0.0377** | 0.8098 | 0.3736 | 0.9914 |
| **C1TI** | **C1IT** | **<0.001** | **0.0065** | **0.0006** | **0.0001** | **<0.001** | **0.0038** | **<0.001** | **<0.001** | **0.0001** | 0.1687 | 0.0168 | 0.0118 |
| **C1SN** | **C1IN** | **0.0002** | 0.0235 | 0.0103 | 0.4512 | **0.0001** | **0.0032** | **0.0004** | **0.0327** | 0.6499 | 0.9149 | 0.8933 | 0.9997 |
| **C1SN** | **C1ST** | **0.0029** | 0.1389 | 0.4842 | 0.5672 | **0.0083** | 0.1195 | 0.1053 | 0.4467 | 0.4640 | 0.9244 | 0.9920 | 0.9154 |
| **C1SN** | **C1IT** | 0.4623 | 0.9863 | 0.9988 | 0.9999 | 0.9370 | 0.9984 | 1.0000 | 0.9949 | 0.9997 | 1.0000 | 0.9999 | 0.8996 |
| **C1IN** | **C1ST** | 0.9986 | 0.9987 | 0.7963 | 1.0000 | 0.9669 | 0.9554 | 0.7784 | 0.9613 | 1.0000 | 1.0000 | 0.9997 | 0.9935 |
| **C1IN** | **C1IT** | 0.2151 | 0.2660 | 0.0801 | 0.1797 | 0.0133 | 0.0330 | **0.0024** | **0.0015** | 0.9075 | 0.9569 | 0.6677 | 0.6055 |
| **C1ST** | **C1IT** | 0.5992 | 0.6652 | 0.8734 | 0.2641 | 0.2431 | 0.4440 | 0.2558 | 0.0819 | 0.7818 | 0.9626 | 0.9191 | 0.1638 |
| **p-values of** |  | **VD Retina C2-ring** | |  |  | **VD SCP C2-ring** | |  |  | **VD DCP C2-ring** | |  |  |
| **pairwise comparisons** | | **BL** | **TS** | **1W** | **6M** | **BL** | **TS** | **1W** | **6M** | **BL** | **TS** | **1W** | **6M** |
| **C2NS** | **C2NI** | 0.3365 | 0.6982 | 0.7462 | 0.9939 | 0.9719 | 0.9879 | 0.9768 | 0.6002 | 0.9891 | 0.9914 | 0.9339 | 0.2444 |
| **C2NS** | **C2TS** | **<0.001** | **<0.001** | **<0.001** | **<0.001** | **<0.001** | **<0.001** | **<0.001** | **<0.001** | **0.0009** | **0.0054** | **0.0003** | **0.0099** |
| **C2NS** | **C2TI** | **<0.001** | **0.0046** | **0.0020** | **<0.001** | **<0.001** | **<0.001** | **<0.001** | **<0.001** | **0.0006** | 0.1762 | **0.0413** | **0.0059** |
| **C2NI** | **C2TS** | **<0.001** | **<0.001** | **<0.001** | **<0.001** | **<0.001** | **<0.001** | **<0.001** | **<0.001** | **0.0031** | **0.0140** | **0.0034** | 0.5532 |
| **C2NI** | **C2TI** | **<0.001** | **0.0001** | **<0.001** | **0.0001** | **<0.001** | **<0.001** | **<0.001** | **<0.001** | **0.0020** | 0.3025 | 0.1728 | 0.4499 |
| **C2TS** | **C2TI** | 0.9091 | 0.2819 | 0.2460 | 0.9086 | 0.9927 | 0.3779 | 0.5267 | 0.9943 | 0.9993 | 0.5844 | 0.5020 | 0.9983 |
| **p-values of** |  | **VD Retina C3-ring** | |  |  | **VD SCP C3-ring** | |  |  | **VD DCP C3-ring** | |  |  |
| **pairwise comparisons** | | **BL** | **TS** | **1W** | **6M** | **BL** | **TS** | **1W** | **6M** | **BL** | **TS** | **1W** | **6M** |
| **C3NS** | **C3NI** | 0.9965 | 0.8216 | 0.9995 | 0.9728 | 0.5608 | 0.9977 | 0.5919 | 0.2673 | 0.9891 | 0.9914 | 0.9339 | 0.2444 |
| **C3NS** | **C3TS** | **0.0038** | 0.0931 | 0.1213 | 0.2870 | **<0.001** | **0.0008** | **0.0003** | **0.0022** | **0.0009** | **0.0054** | **0.0003** | **0.0099** |
| **C3NS** | **C3TI** | 0.0694 | 0.6592 | 0.7548 | 0.7787 | **<0.001** | **0.0202** | **0.0008** | **0.0055** | **0.0006** | 0.1762 | **0.0413** | **0.0059** |
| **C3NI** | **C3TS** | **0.0080** | **0.0079** | 0.0933 | 0.5293 | **0.0006** | **0.0004** | **0.0288** | 0.2481 | **0.0031** | 0.0140 | **0.0034** | 0.5532 |
| **C3NI** | **C3TI** | 0.1155 | 0.1839 | 0.6874 | 0.9527 | **0.0009** | **0.0114** | **0.0489** | 0.3793 | **0.0020** | 0.3025 | 0.1728 | 0.4499 |
| **C3TS** | **C3TI** | 0.7885 | 0.6489 | 0.6330 | 0.8501 | 0.9999 | 0.7900 | 0.9983 | 0.9952 | 0.9993 | 0.5844 | 0.5020 | 0.9983 |

**Supplementary Table S3A**: Results of the vessel density (VD) are presented as p-values for all retinal OCTA slabs (Retina, SCP and DCP) and all four follow-up visits (BL, TS, 1W, 6M) for all pairwise comparisons between all subsections of the inner ring, outer ring, C1-, C2 and C3-ring as indicated with the ETDRS grid in Figure 1. Significant p-values are labelled in bold.

| **p-values of** |  | **PD Retina Inner Ring** | |  |  | **PD SCP Inner Ring** | |  |  | **PD DCP Inner Ring** | |  |  |
| --- | --- | --- | --- | --- | --- | --- | --- | --- | --- | --- | --- | --- | --- |
| **pairwise comparisons** | | **BL** | **TS** | **1W** | **6M** | **BL** | **TS** | **1W** | **6M** | **BL** | **TS** | **1W** | **6M** |
| **IN** | **IS** | 0.9811 | 0.6890 | 0.8729 | 0.9252 | 1.0000 | 0.9149 | 0.9579 | 0.9995 | 0.9474 | 0.6864 | 0.8676884 | 0.9785156 |
| **IN** | **IT** | 0.8557 | 0.9593 | 1.0000 | 0.9719 | 0.5719 | 0.9714 | 0.8116 | 0.6217 | 0.9394 | 0.9916 | 0.9970589 | 0.9888164 |
| **IN** | **II** | 0.8563 | 0.3629 | 0.3924 | 0.9978 | 0.9266 | 0.7685 | 0.4503 | 1.0000 | 0.9705 | 0.6684 | 0.811978 | 0.9909378 |
| **IS** | **IT** | 0.6403 | 0.9315 | 0.8658 | 0.7156 | 0.5690 | 0.6942 | 0.5041 | 0.5494 | 0.6757 | 0.8467 | 0.9415478 | 0.8905248 |
| **IS** | **II** | 0.9760 | 0.9526 | 0.8464 | 0.9720 | 0.9280 | 0.9892 | 0.7645 | 0.9998 | 0.9996 | 1.0000 | 0.9994479 | 0.999615 |
| **IT** | **II** | 0.3824 | 0.6717 | 0.3830 | 0.9250 | 0.2266 | 0.4948 | 0.0837 | 0.6035 | 0.7370 | 0.8327 | 0.903332 | 0.9274755 |
| **p-values of** |  | **PD Retina Outer Ring** | |  |  | **PD SCP Outer Ring** | |  |  | **PD DCP Outer Ring** | |  |  |
| **pairwise comparisons** | | **BL** | **TS** | **1W** | **6M** | **BL** | **TS** | **1W** | **6M** | **BL** | **TS** | **1W** | **6M** |
| **ON** | **OS** | 0.2079 | 0.6759 | 0.2998 | 0.5440 | **0.0068** | 0.2662 | **0.0294** | 0.1372 | 0.7026 | 0.9976 | 0.7205 | 0.8647 |
| **ON** | **OT** | **<0.001** | **0.0034** | **0.0010** | **0.0063** | **<0.001** | **0.0002** | **<0.001** | **0.0001** | **0.0071** | 0.2781 | 0.0516 | 0.1527 |
| **ON** | **OI** | 0.8858 | 0.9999 | 0.9962 | 0.5404 | 0.2073 | 0.8175 | 0.3271 | 0.2467 | 0.7997 | 1.0000 | 0.9875 | 0.6884 |
| **OS** | **OT** | **0.0003** | 0.0970 | 0.1863 | 0.1919 | **0.0009** | 0.0925 | 0.1153 | 0.0705 | 0.1413 | 0.3788 | 0.4324 | 0.5356 |
| **OS** | **OI** | 0.6140 | 0.6356 | 0.4227 | 1.0000 | 0.5765 | 0.7836 | 0.7089 | 0.9902 | 0.9983 | 0.9979 | 0.8900 | 0.9879 |
| **OT** | **OI** | **<0.001** | **0.0027** | **0.0024** | 0.1940 | **<0.001** | **0.0061** | **0.0054** | **0.0327** | 0.0965 | 0.2824 | 0.1161 | 0.7378 |
| **p-values of** |  | **PD Retina C1-ring** | |  |  | **PD SCP C1-ring** | |  |  | **PD DCP C1-ring** | |  |  |
| **pairwise comparisons** | | **BL** | **TS** | **1W** | **6M** | **BL** | **TS** | **1W** | **6M** | **BL** | **TS** | **1W** | **6M** |
| **C1NS** | **C1NI** | 0.1571 | 0.3685 | 0.2951 | 0.9991 | 0.1024 | 0.3371 | 0.2449 | 0.9946 | 0.9962 | 0.9986 | 0.9996 | 0.9919 |
| **C1NS** | **C1TS** | **<0.001** | **<0.001** | **<0.001** | **<0.001** | **<0.001** | **<0.001** | **<0.001** | **<0.001** | **<0.001** | **0.0014** | **0.0001** | **0.0007** |
| **C1NS** | **C1TI** | **<0.001** | **0.0008** | **0.0004** | **<0.001** | **<0.001** | **<0.001** | **<0.001** | **<0.001** | **<0.001** | **0.0169** | **0.0008** | **0.0002** |
| **C1NS** | **C1SN** | 0.9889 | 0.9996 | 0.9998 | 0.8944 | 0.9983 | 0.9992 | 0.9997 | 0.4196 | 0.8143 | 0.9992 | 0.9269 | 0.2277 |
| **C1NS** | **C1IN** | **0.0093** | 0.1226 | 0.0568 | **0.0107** | **<0.001** | **0.0004** | **0.0002** | **<0.001** | **0.0378** | 0.6764 | 0.1984 | 0.0572 |
| **C1NS** | **C1ST** | 0.0785 | 0.4072 | 0.7926 | 0.0228 | **0.0016** | **0.0311** | 0.0655 | **0.0011** | **0.0177** | 0.5283 | 0.4248 | **0.0052** |
| **C1NS** | **C1IT** | 0.9805 | 0.9988 | 1.0000 | 0.9913 | 0.8524 | 0.9443 | 0.9985 | 0.9524 | 0.4793 | 0.9915 | 0.9834 | 0.9259 |
| **C1NI** | **C1TS** | **<0.001** | **<0.001** | **<0.001** | **<0.001** | **<0.001** | **<0.001** | **<0.001** | **<0.001** | **<0.001** | **0.0001** | **<0.001** | **0.0160** |
| **C1NI** | **C1TI** | **<0.001** | **<0.001** | **<0.001** | **<0.001** | **<0.001** | **<0.001** | **<0.001** | **<0.001** | **<0.001** | **0.0016** | **0.0001** | **0.0049** |
| **C1NI** | **C1SN** | 0.6682 | 0.7170 | 0.5961 | 0.5617 | **0.0149** | 0.0991 | 0.0743 | 0.0838 | 0.3424 | 0.9342 | 0.6717 | 0.7371 |
| **C1NI** | **C1IN** | **<0.001** | **<0.001** | **<0.001** | **0.0011** | **<0.001** | **<0.001** | **<0.001** | **<0.001** | **0.0030** | 0.2711 | 0.0550 | 0.3752 |
| **C1NI** | **C1ST** | **<0.001** | **0.0005** | **0.0033** | **0.0029** | **<0.001** | **<0.001** | **<0.001** | **<0.001** | **0.0011** | 0.1735 | 0.1593 | 0.0731 |
| **C1NI** | **C1IT** | **0.0092** | 0.1081 | 0.2409 | 0.8506 | 0.0008 | 0.0183 | 0.0568 | 0.5574 | 0.1133 | 0.8361 | 0.8450 | 0.9999 |
| **C1TS** | **C1TI** | 0.9999 | 0.9436 | 0.9987 | 0.9999 | 0.9627 | 0.9329 | 0.9985 | 0.9999 | 1.0000 | 0.9980 | 0.9997 | 1.0000 |
| **C1TS** | **C1SN** | **<0.001** | **<0.001** | **<0.001** | **0.0001** | **<0.001** | **<0.001** | **<0.001** | **0.0001** | **0.0001** | **0.0137** | **0.0140** | 0.6872 |
| **C1TS** | **C1IN** | **0.0005** | 0.1490 | 0.5073 | 0.1993 | **0.0075** | 0.6908 | 0.6892 | 0.6188 | 0.0591 | 0.2844 | 0.3789 | 0.9235 |
| **C1TS** | **C1ST** | **<0.001** | **0.0325** | **0.0144** | 0.1586 | **0.0001** | 0.0889 | **0.0241** | 0.1083 | 0.1120 | 0.4308 | 0.1693 | 0.9999 |
| **C1TS** | **C1IT** | **<0.001** | **0.0001** | **0.0001** | **<0.001** | **<0.001** | **<0.001** | **<0.001** | **<0.001** | **0.0011** | **0.0345** | **0.0063** | 0.0560 |
| **C1TI** | **C1SN** | **<0.001** | **0.0001** | **<0.001** | **0.0007** | **<0.001** | **0.0006** | **<0.001** | **0.0003** | **<0.001** | 0.0996 | 0.0642 | 0.4670 |
| **C1TI** | **C1IN** | **0.0028** | 0.8283 | 0.8816 | 0.4122 | 0.1807 | 0.9997 | 0.9628 | 0.8477 | **0.0185** | 0.7146 | 0.7073 | 0.7761 |
| **C1TI** | **C1ST** | **0.0002** | 0.4662 | 0.0939 | 0.3434 | **0.0089** | 0.7293 | 0.1412 | 0.2531 | **0.0395** | 0.8478 | 0.4298 | 0.9955 |
| **C1TI** | **C1IT** | **<0.001** | **0.0108** | **0.0009** | **<0.001** | **<0.001** | **0.0067** | **<0.001** | **<0.001** | **0.0002** | 0.1937 | **0.0325** | **0.0203** |
| **C1SN** | **C1IN** | **0.0003** | **0.0311** | **0.0131** | 0.3995 | **0.0003** | **0.0043** | **0.0016** | 0.0560 | 0.7283 | 0.9495 | 0.9084 | 0.9997 |
| **C1SN** | **C1ST** | **0.0046** | 0.1555 | 0.4887 | 0.5278 | **0.0174** | 0.1565 | 0.2229 | 0.5028 | 0.5691 | 0.8787 | 0.9889 | 0.9166 |
| **C1SN** | **C1IT** | 0.6049 | 0.9572 | 0.9990 | 0.9997 | 0.9946 | 0.9991 | 1.0000 | 0.9754 | 0.9996 | 1.0000 | 1.0000 | 0.9227 |
| **C1IN** | **C1ST** | 0.9976 | 0.9992 | 0.8291 | 1.0000 | 0.9711 | 0.9479 | 0.7682 | 0.9769 | 1.0000 | 1.0000 | 0.9999 | 0.9942 |
| **C1IN** | **C1IT** | 0.1583 | 0.4404 | 0.0931 | 0.1300 | **0.0071** | **0.0355** | **0.0034** | **0.0012** | 0.9526 | 0.9889 | 0.7924 | 0.6451 |
| **C1ST** | **C1IT** | 0.5393 | 0.8159 | 0.8714 | 0.2086 | 0.1569 | 0.4824 | 0.3130 | 0.0543 | 0.8762 | 0.9601 | 0.9513 | 0.1924 |
| **p-values of** |  | **PD Retina C2-ring** | |  |  | **PD SCP C2-ring** | |  |  | **PD DCP C2-ring** | |  |  |
| **pairwise comparisons** | | **BL** | **TS** | **1W** | **6M** | **BL** | **TS** | **1W** | **6M** | **BL** | **TS** | **1W** | **6M** |
| **C2NS** | **C2NI** | 0.6369 | 0.8389 | 0.9070 | 0.9390 | 0.9964 | 0.9994 | 0.9974 | 0.4725 | 0.9906 | 0.9869 | 0.9401 | 0.2795 |
| **C2NS** | **C2TS** | **<0.001** | **<0.001** | **<0.001** | **<0.001** | **<0.001** | **<0.001** | **<0.001** | **<0.001** | **0.0011** | **0.0060** | **0.0005** | **0.0139** |
| **C2NS** | **C2TI** | **<0.001** | **0.0016** | **0.0011** | **<0.001** | **<0.001** | **<0.001** | **<0.001** | **<0.001** | **0.0009** | 0.1529 | 0.0540 | **0.0078** |
| **C2NI** | **C2TS** | **<0.001** | **<0.001** | **<0.001** | **<0.001** | **<0.001** | **<0.001** | **<0.001** | **<0.001** | **0.0034** | **0.0179** | **0.0042** | 0.5764 |
| **C2NI** | **C2TI** | **<0.001** | **<0.001** | **0.0001** | **0.0001** | **<0.001** | **<0.001** | **<0.001** | **<0.001** | **0.0027** | 0.2909 | 0.2023 | 0.4574 |
| **C2TS** | **C2TI** | 0.8343 | 0.2998 | 0.2643 | 0.9355 | 0.9802 | 0.4292 | 0.5399 | 0.9951 | 0.9999 | 0.6494 | 0.4871 | 0.9975 |
| **p-values of** |  | **PD Retina C3-ring** | |  |  | **PD SCP C3-ring** | |  |  | **PD DCP C3-ring** | |  |  |
| **pairwise comparisons** | | **BL** | **TS** | **1W** | **6M** | **BL** | **TS** | **1W** | **6M** | **BL** | **TS** | **1W** | **6M** |
| **C3NS** | **C3NI** | 0.9785 | 0.8915 | 1.0000 | 0.9553 | 0.4554 | 0.9997 | 0.6011 | 0.2815 | 0.9999 | 0.9981 | 0.9979 | 0.9738 |
| **C3NS** | **C3TS** | **0.0020** | 0.0621 | 0.0721 | 0.2880 | **<0.001** | **0.0008** | **0.0004** | **0.0033** | 0.7923 | 0.7000 | 0.9980 | 0.9717 |
| **C3NS** | **C3TI** | **0.0496** | 0.5569 | 0.6072 | 0.7618 | **<0.001** | **0.0186** | **0.0007** | **0.0067** | 1.0000 | 1.0000 | 0.9992 | 0.7679 |
| **C3NI** | **C3TS** | **0.0082** | **0.0076** | 0.0731 | 0.5823 | **0.0009** | **0.0005** | **0.0286** | 0.2923 | 0.7569 | 0.5896 | 1.0000 | 0.8250 |
| **C3NI** | **C3TI** | 0.1301 | 0.1809 | 0.6108 | 0.9645 | **0.0015** | **0.0139** | **0.0435** | 0.3984 | 0.9996 | 0.9969 | 0.9898 | 0.5115 |
| **C3TS** | **C3TI** | 0.7622 | 0.6465 | 0.6468 | 0.8647 | 0.9995 | 0.8106 | 0.9993 | 0.9979 | 0.8184 | 0.7227 | 0.9903 | 0.9518 |

**Supplementary Table S3B**: Results of the perfusion density (PD) are presented as p-values for all retinal OCTA slabs (Retina, SCP and DCP) and all four follow-up visits (BL, TS, 1W, 6M) for all pairwise comparisons between all subsections of the inner ring, outer ring, C1-, C2 and C3-ring as indicated with the ETDRS grid in Figure 1. Significant p-values are labelled in bold.

| **p-values of** |  | **CFI** |  |  |  | **CPD** |  |  |  |
| --- | --- | --- | --- | --- | --- | --- | --- | --- | --- |
| **pairwise comparisons** | | **BL** | **TS** | **1W** | **6M** | **BL** | **TS** | **1W** | **6M** |
| **T** | **S** | 0.9996 | 0.9996 | 0.9996 | 0.9998 | **0.0054** | 0.3229 | 0.8109 | 0.0678 |
| **T** | **N** | 0.9715 | 0.9700 | 0.9829 | 0.7423 | 0.1170 | **0.0436** | 0.8984 | **0.0310** |
| **T** | **I** | 1.0000 | 0.9952 | 0.9497 | 0.9371 | 0.1920 | 0.3425 | 0.9442 | 0.2228 |
| **S** | **N** | 0.9872 | 0.9473 | 0.9661 | 0.6938 | 0.6231 | 0.7557 | 0.9974 | 0.9871 |
| **S** | **I** | 0.9991 | 0.9990 | 0.9199 | 0.9100 | 0.4681 | 1.0000 | 0.9873 | 0.9334 |
| **N** | **I** | 0.9656 | 0.9055 | 0.9982 | 0.9725 | 0.9944 | 0.7628 | 0.9990 | 0.7908 |

**Supplementary Table S3C**: Results of the capillary flux index (CFI, left) and capillary perfusion density (CPD, right) are presented as p-values for all four follow-up visits (BL, TS, 1W, 6M) for all pairwise comparisons between all subsections of the peripapillary ring as indicated with the grid in Figure 2. Significant p-values are labelled in bold.
